# Supplementary material for: Inference and analysis of cell-cell communication using CellChat
Source: Nat Commun. 2021 Feb 17;12:1088. doi: 10.1038/s41467-021-21246-9 (PMC7889871; doi:10.1038/s41467-021-21246-9)
Supplement: Supplementary file 1 — Supplementary Information [file 41467_2021_21246_MOESM1_ESM.pdf]

Supplementary Information

Inference and analysis of cell-cell communication using CellChat

Suoqin Jin, Christian F. Guerrero-Juarez, Lihua Zhang, Ivan Chang, Raul Ramos, Chen-Hsiang Kuan, Peggy Myung, Maksim V. Plikus[#], Qing Nie[#]

This file includes the following subsections:

- Supplementary Notes
  - Supplementary Note 1
    - Detailed steps for collecting ligand–receptor interactions in CellChatDB
    - Update CellChatDB by adding user-defined ligand-receptor pairs
    - Comparison of CellChatDB with KEGG database
    - Comparison of CellChatDB with other existing ligand-receptor databases
  - Supplementary Note 2
    - Communication pattern analysis using different number of patterns
    - Influence of cell clustering on the inferred cell-cell communication network
  - Supplementary Note 3
    - Details of method comparisons
    - Evaluation metrics
  - References
- Supplementary Figures 1-17
- Supplementary Table 1
- Supplementary Table 2

Supplementary Notes

Supplementary Note 1

Detailed steps for collecting ligand–receptor interactions in CellChatDB

CellChatDB is a database of literature-supported ligand-receptor interactions in mouse and human. The majority of ligand-receptor interactions were manually curated on the basis of KEGG signaling pathway database, and additional signaling molecular interactions were gathered from recent peer-reviewed studies. In particular, the detailed steps for collecting ligand-receptor interactions in mouse are as follows.

Step 1: We collected the list of all major signaling pathway families, which are related to "Signal Transduction" and "Signaling Molecules and Interaction" (https://www.genome.jp/kegg/pathway.html) in KEGG pathway database.

Step 2: We manually curated ligand-receptor interactions by reviewing all relevant KEGG pathway maps. For each map, e.g., TGFβ signaling pathway (Supplementary Fig. 1a), each signaling molecule was classified as one of six categories: ligand, receptor, agonist, antagonist, co-stimulatory and co-inhibitory membrane-bound receptors. Signaling role of each molecule is clearly indicated in KEGG pathway maps. Note that agonist, antagonist, co-stimulatory and co-inhibitory receptors are considered as cofactor molecules, which only modulate ligand-receptor mediated signaling strength, and do not produce new ligand-receptor pairs. After a ligand-receptor pair was chosen, we determined its co-factor molecules. For example, for Tgfb1 ligand (green box) and its receptor complex Tgfbr1/Tgfbr2 (pink box) (new Supplementary Fig. 1a), Thbs1 (light orange box) is found to be an agonist because Thbs1 inhibits Ltbp1 and Lrbp1 inhibits the ligand Tgfb1, implying a positive regulation of Tgfb1 by Thbs1. For this case, antagonists include Ltbp1, Lefty1, Fmod, Dcn (orange box) because they are negative regulators of Tgfb1. Co-inhibitory receptor is Bambi (purple) because of its negative role as a membrane-bound receptor. Similarly, we collected other ligand-receptor pairs as well as cofactors by reviewing all available KEGG pathway maps. Supplementary Figure 1a shows several other examples of ligand-receptor pairs and how we defined ligands, (multi-subunit) receptors and cofactors. Note that we did not consider ligand-co-receptors pairs, agonist-receptor pairs and antagonist-receptor pairs because these co-factors usually either enhance or attenuate the main ligand-receptor interaction to modulate signaling.

Step 3: We carefully reviewed several signaling molecule/compound families in the KEGG database, including Cytokine Receptors (ko04050), Cytokines and Growth Factors (ko04052), and Bioactive Peptides (br08005), to ensure that these ligands/receptors are included in the curated ligand-receptor pairs in Step 2.

Step 4: We collected additional ligand-receptor interactions by reviewing the known signaling pathway families in recent peer-reviewed studies. For example, for TGFβ signaling pathway, we collected nine additional ligand-receptor pairs from two prominent papers[1, 2]. A recent experimental study showed that TGFβ uses a novel mode of receptor activation (i.e., TGFBR1 and ACVR1) to phosphorylate SMAD1/5[1]. Another study also showed that TGFβ ligands can bind to type I receptor ACVR1B/ACVR1C[2]. The evidence for each literature-supported ligand-receptor interaction is provided in CellChatDB. Together, mouse CellChatDB contains 2,021 validated molecular interactions, including 60% of paracrine/autocrine signaling interactions, 21% of extracellular matrix (ECM)-receptor interactions and 19% of cell-cell contact interactions.

To collect signaling interactions in human, mouse gene symbols in CellChatDB were mapped to human orthologues using the human orthologue information via MGI (http://www.informatics.jax.org/homology.shtml). We also manually added signaling interactions specific to human, including CXCL8, CCL13, CCL14, CCL15, CCL16, CCL23, XCL2, IFNW1, IL1F7, IL26, IL29. In sum, human CellChatDB contains 1,939 validated molecular interactions, including 61.8% of paracrine/autocrine signaling interactions, 21.7% of extracellular matrix (ECM)-receptor interactions and 16.5% of cell-cell contact interactions.

Update CellChatDB by adding user-defined ligand-receptor pairs

Users can update CellChatDB by adding their own curated ligand-receptor pairs. To do so, the format of the users' lists must be compatible with the input files of CellChatDB. Users can update CellChatDB by submitting their lists via a pull request at Github (https://github.com/sqjin/CellChat) or following the four steps as follows.

Step 1, extract the database information in CellChatDB and then save them in a local computer, including four files: 'geneInfo.csv', 'interaction_input_CellChat.csv', 'complex_input_CellChat.csv', 'and cofactor_input_CellChat.csv'. Users can do it by running the following codes in Rstudio:

```
library(CellChat)
options(stringsAsFactors = FALSE)
CellChatDB <- CellChatDB.mouse # CellChatDB <- CellChatDB.human
interaction_input <- CellChatDB$interaction
complex_input <- CellChatDB$complex
cofactor_input <- CellChatDB$cofactor
geneInfo <- CellChatDB$geneInfo
write.csv(interaction_input, file = "interaction_input_CellChatDB.csv")
write.csv(complex_input, file = "complex_input_CellChatDB.csv")
write.csv(cofactor_input, file = "cofactor_input_CellChatDB.csv")
write.csv(geneInfo, file = "geneInfo_input_CellChatDB.csv")
```

Step 2, update these four .csv files by adding users' curated ligand-receptor pairs. The main file is 'interaction_input_CellChatDB.csv'. Users can first update the ligands, receptors and co-factors in the corresponding columns in 'interaction_input_CellChatDB.csv'. Users can then update *"complex_input_CellChatDB.csv"* and "cofactor_input_CellChatDB.csv" if any ligand complex, receptor complex and cofactors are updated. Users need to make sure that user-defined complex/cofactor names are the same in 'interaction_input_CellChatDB.csv' and *"complex_input_CellChatDB.csv",* 'interaction_input_CellChatDB.csv' and *"* cofactor_input_CellChatDB*.csv". "geneInfo_input_CellChatDB.csv"* contains all gene information in mouse and it should have a column named 'Symbol', which does not need to be changed when updating CellChatDB.

Step 3, update CellChatDB once updating the four .csv files. Users can do it by running the following codes in Rstudio:

```
options(stringsAsFactors = FALSE)
interaction_input <- read.csv(file = 'interaction_input_CellChatDB.csv', row.names = 1)
complex_input <- read.csv(file = 'complex_input_CellChatDB.csv', row.names = 1)
cofactor_input <- read.csv(file = 'cofactor_input_CellChatDB.csv', row.names = 1)
geneInfo <- read.csv(file = ' geneInfo_input_CellChatDB.csv', row.names = 1)
CellChatDB <- list()
CellChatDB$interaction <- interaction_input
CellChatDB$complex <- complex_input
CellChatDB$cofactor <- cofactor_input
CellChatDB$geneInfo <- geneInfo
```

Step 4, re-build CellChat package by updating the database as follows

```
setwd("/Users/USERS/Downloads/CellChat-master") # This is the folder of CellChat package downloaded from Github
CellChatDB.mouse <- CellChatDB
devtools::use_data(CellChatDB.mouse, overwrite = TRUE)
```

Comparison of CellChatDB with KEGG database

Although KEGG database contains the ligand-receptor interaction information, such information is not "ready-to-use" when developing a systematical approach for inferring cell-cell communication. By incorporating carefully reviewed KEGG pathway maps and recent peer-reviewed studies, CellChatDB provides the following added value.

- It collects ligand-receptor pairs from KEGG signaling pathway maps.
- It categories signaling molecules in each KEGG pathway map into different groups based on their roles in ligand-receptor interaction: agonist, antagonist, co-stimulatory receptor and co-inhibitory receptor.

- It arranges all collected information into a structured data format, allowing for easy computational analysis and provides new community resource to develop a systematical approach for cell-cell communication analysis.
- It adds ligand-receptor interactions that are not in KEGG database through careful review of known signaling pathways in recent peer-reviewed studies.

Comparison of CellChatDB with other existing ligand-receptor databases

CellChatDB incorporates not only multi-subunit structure of ligand-receptor (L-R) complexes but also soluble and membrane-bound stimulatory and inhibitory cofactors, leading to a more comprehensive database than other existing ligand-receptor databases. We quantitatively showed the differences and the strengths of CellChatDB in comparison to other existing analogous databases, including CellTalkDB[3], CellPhoneDB[4], iTALK[5], SingleCellSignalR[6], Ramilowski2015[7], NicheNet[8] and ICELLNET[9]. Among these databases, only CellChatDB and CellTalkDB contain ligand-receptor interactions in mouse. Therefore, we first performed comparison of the human version of CellChatDB with the above databases by counting the number of L-R pairs, L-R pairs with multi-subunits, and L-R pairs with cofactors (e.g., agonist, antagonist, co-stimulatory and co-inhibitory receptors) (Supplementary Fig. 1b). First, the number of L-R pairs in CellChatDB (i.e., 1,939 pairs) is higher than both in CellPhoneDB and ICELLNET, but lower than in other five databases, among which NicheNet has the largest number of L-R pairs (i.e., 12,651 pairs). Second, the number of L-R pairs with multi-subunits in CellChatDB (i.e., 928 pairs) is twice larger than in CellPhoneDB and three times larger than in ICELLNET, whereas other four databases do not consider L-R pairs with multi-subunits. Third, only CellChatDB contains L-R pairs with cofactors. Moreover, we performed comparison of the mouse version of CellChatDB with CellTalkDB, which showed comparable number of L-R pairs (2021 vs. 2033). However, CellTalkDB does not consider the L-R pairs with multi-subunits as well as the cofactors. Taking into an account subunit structure of ligands and receptors is essential because cell-cell communication often relies on multi-subunit protein complexes. Cofactors also modulate cell-cell communication both positively and negatively for certain signaling pathways. Therefore, CellChatDB provides an important resource for identifying biologically meaningful cell-cell communication.

Supplementary Note 2

Communication pattern analysis using different number of patterns

In the examples of communication pattern analysis on skin wound dataset and E14.5 embryonic skin pseudotime dataset, we showed how different cell populations coordinate with each other and how they coordinate with certain signaling pathways to drive and respond to signals by predicting five latent patterns (Fig. 2g-h and Fig. 3g-h). Here we also employed unsupervised method to infer the number of patterns based on two metrics including Cophenetic and Silhouette[10] (see Methods). A suitable number of patterns is the one at which Cophenetic and Silhouette values begin to suddenly drop. By applying these two metrics to the two aforementioned datasets, we found that the inferred number of patterns was ranging from 4 to 6 (Supplementary Fig. 15a-b). For the outgoing communication patterns in the wound dataset and the incoming communication patterns in the E14.5 pseudotime dataset, these two metrics predicted that the number of patterns was 4 and 6, respectively. Comparing to the five outgoing communication patterns in the wound dataset (Fig. 2g), the four outgoing communication patterns merged two fibroblast-related patterns into one pattern. Specifically, FIB-H and other three fibroblast subpopulations (FIB-D, FIB-F and FIB-I) that were originally associated with two different patterns (Fig. 2g) were now associated with a single pattern (Supplementary Fig. 15d). For the E14.5 embryonic DC_Placode dataset with four spatially colocalized cell populations, the two metrics predicted that the number of incoming communication patterns was two (Supplementary Fig. 15c). Compared to the three incoming communication patterns (Fig. 4f), the two incoming patterns merged the pre-DC-enriched pattern with the DC-enriched pattern (Supplementary Fig. 15e). In another words, pre-DC and DC that were originally enriched in two different patterns (Fig. 4f) were now enriched in a single pattern (Supplementary Fig. 15e). Different numbers of patterns provide different resolution to uncovering the coordinated responses among different cell types. CellChat R package provides a visual representation of Cophenetic and Silhouette metrics for a range of the numbers of patterns to enable users select the optimal setting for the number of patterns present in the dataset. Of note, we use matrix decomposition instead of tensor decomposition approach to perform the communication pattern analysis, which is partly because the tensor decomposition algorithm is not applicable to the following two situations: (1) numbers of outgoing and incoming patterns could be different, and (2) coordinated signaling pathways could be different for secreting and receiving cells.

Influence of cell clustering on the inferred cell-cell communication network

To explore how cell clustering results may affect the inferred cell-cell communication network, we used an example of E14.5 mouse embryonic skin dataset with four spatially colocalized cell subpopulations. First, we assessed how cell clustering affects the inferred interactions if the number of cell clusters remains the same. We used two different choices of parameters (e.g., different number of highly variable genes and principle components) to produce two different clustering results while keeping the number of cell clusters unchanged. The Jaccard similarities between these two new clustering results and our original clustering

result were 0.91 and 0.83, respectively. We then re-run CellChat analysis and found that all of the inferred interactions from our original clustering result were also predicted using these two newly added clustering results (Supplementary Fig. 16). Second, we used another choice of parameters to produce different number of cell clusters, leading to three subpopulations: Placode, preDC and DC. Applying CellChat to these three subpopulations, we found that 88% of interactions inferred from our original clustering result were also predicted using new clustering result (Supplementary Fig. 16). These results were not surprising for the following two reasons: 1) different number of cell clusters likely produces different ligand-receptor interactions because of the different resolution of cell-cell communication analysis; 2) the uncertainty of the clustering results will not likely give different ligand-receptor interactions because cell-cell communication is inferred at the clustering level and some degree of uncertainty will not likely affect the estimation of the average gene expression of the cell clusters.

Supplementary Note 3

Details of method comparisons

Details for running SingleCellSignalR. SingleCellSignalR v0.0.1.4[6] was used for inferring intercellular communications in scRNA-seq datasets (https://github.com/SCA-IRCM/SingleCellSignalR_v1). To infer both "autocrine" and "paracrine" signaling, we used the “cell_signaling” function with the “int.type” parameter being "autocrine" and the “species” parameter being "mus musculus".

Details for running iTALK. iTALK v0.1.0[5] was used for inferring intercellular communications in scRNA-seq datasets (https://github.com/Coolgenome/iTALK). Since iTALK only provides ligand-receptor database for human, we first mapped gene symbols from mouse to human and then ran the default iTALK workflow provided in https://github.com/Coolgenome/iTALK/blob/master/example/example_code.r.

Details for running CellPhoneDB. CellPhoneDB v2.0.0[4] was used for inferring intercellular communications in scRNA-seq datasets (https://github.com/Teichlab/cellphonedb). Since CellPhoneDB only provides ligand-receptor database for human, we first mapped the gene symbols from mouse to human and then ran “cellphonedb method statistical_analysis metadata.txt counts.txt --iterations=100 --threads=2”.

Evaluation metrics

We evaluated the robustness of inferred interactions from subsampled datasets using three measures, including true positive rate (TPR), false positive rate (FPR) and accuracy (ACC) by comparing the subsampled dataset with the original dataset. They are defined as follows:

$$TPR = \frac{TP}{TP + FN} \quad (1)$$

$$FPR = \frac{FP}{TN + FP} \quad (2)$$

$$ACC = \frac{TP + TN}{TP + TN + FP + FN} \quad (3)$$

where TP (true positive) is the number of interactions inferred from the subsampled dataset matched by the interactions inferred from the original dataset, FP (false positive) is the total number of interactions of the subsampled dataset minus TP, TN (true negative) is the number of interactions not in both the subsampled dataset and the original dataset, and FN (false negative) is the number of interaction of the original dataset that are not matched by the subsampled dataset.

Supplementary Reference

1. Ramachandran, A. et al. TGF-beta uses a novel mode of receptor activation to phosphorylate SMAD1/5 and induce epithelial-to-mesenchymal transition. *Elife* **7** (2018).
2. Hata, A. & Chen, Y.G. TGF-beta Signaling from Receptors to Smads. *Cold Spring Harb Perspect Biol* **8** (2016).
3. Shao, X. et al. CellTalkDB: a manually curated database of ligand-receptor interactions in humans and mice. *Brief Bioinform* (2020).
4. Efremova, M., Vento-Tormo, M., Teichmann, S.A. & Vento-Tormo, R. CellPhoneDB: inferring cell–cell communication from combined expression of multi-subunit ligand–receptor complexes. *Nature Protocols* (2020).
5. Wang, Y. et al. iTALK: an R Package to Characterize and Illustrate Intercellular Communication. *bioRxiv*, 507871 (2019).
6. Cabello-Aguilar, S. et al. SingleCellSignalR: inference of intercellular networks from single-cell transcriptomics. *Nucleic Acids Res* **pii**, gkaa183 (2020).
7. Ramilowski, J.A. et al. A draft network of ligand-receptor-mediated multicellular signalling in human. *Nat Commun* **6**, 7866 (2015).
8. Browaeys, R., Saelens, W. & Saeys, Y. NicheNet: modeling intercellular communication by linking ligands to target genes. *Nat Methods* **17**, 159-162 (2020).
9. Noël, F. et al. ICELLNET: a transcriptome-based framework to dissect intercellular communication. *bioRxiv*, 2020.2003.2005.976878 (2020).
10. Gaujoux, R. & Seoighe, C. A flexible R package for nonnegative matrix factorization. *BMC Bioinformatics* **11**, 367 (2010).

Supplementary Figures

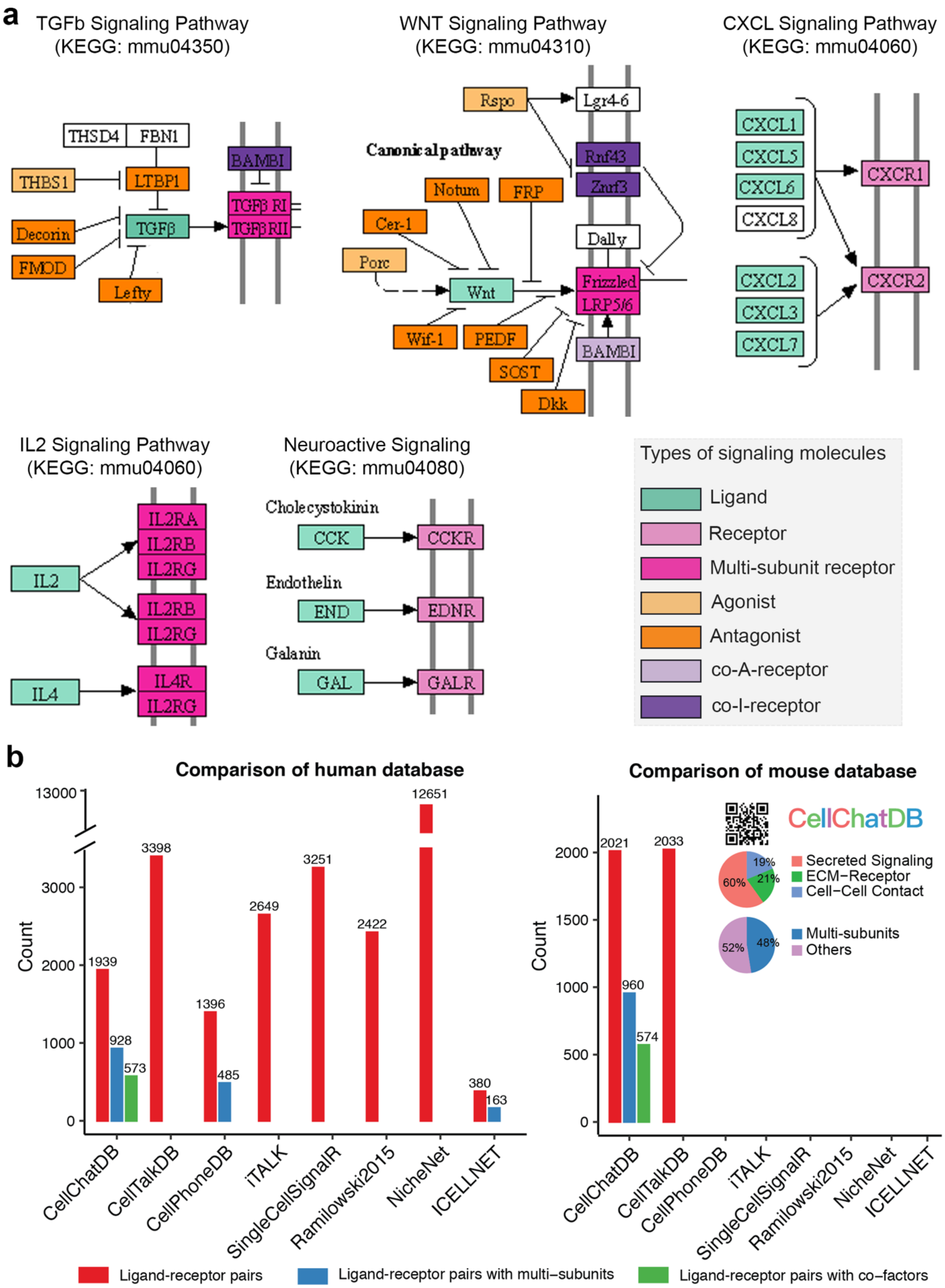

Supplementary Figure 1: Details for manually curating CellChatDB and comparisons of CellChatDB with seven other known databases. (a) Examples of signaling pathways showing how we defined ligands, receptors, multi-subunit receptors, soluble agonist, soluble antagonist, co-A-receptor (co-stimulatory receptor) and co-I-receptor (co-inhibitory receptor) based on the KEGG pathway maps. Each panel shows one mouse KEGG pathway map, in which signaling molecules are colored based on their signaling roles on the ligand-receptor interaction. Note that we only show a part of each KEGG pathway map and CXCL8 is not a mouse gene. **(b)** Comparison of CellChatDB with seven other known databases by counting the number of L-R pairs, L-R pairs with multi-subunits, and L-R pairs with co-factors (e.g., soluble agonist, antagonist, co-stimulatory and co-inhibitory receptors). Only CellChatDB and CellTalkDB contain ligand-receptor interactions in mouse. The mouse version of CellChatDB contains 2,021 validated interactions, including 60% of secreting interactions. In addition, 48% of the interactions involve heteromeric molecular complexes.

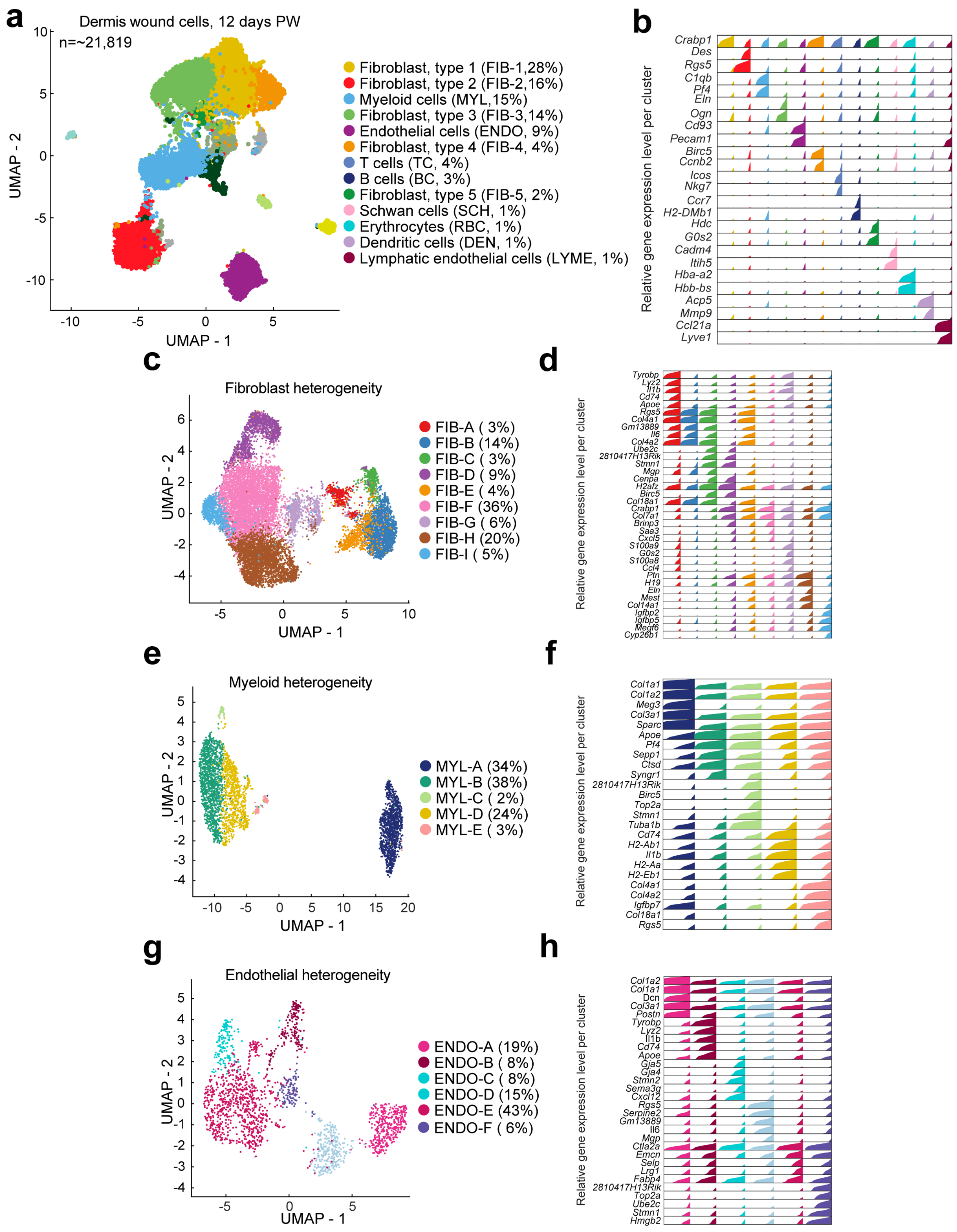
a
Dermis wound cells, 12 days PW
n=~21,819
UMAP - 1
UMAP - 2
Fibroblast, type 1 (FIB-1,28%)
Fibroblast, type 2 (FIB-2,16%)
Myeloid cells (MYL,15%)
Fibroblast, type 3 (FIB-3,14%)
Endothelial cells (ENDO, 9%)
Fibroblast, type 4 (FIB-4, 4%)
T cells (TC, 4%)
B cells (BC, 3%)
Fibroblast, type 5 (FIB-5, 2%)
Schwan cells (SCH, 1%)
Erythrocytes (RBC, 1%)
Dendritic cells (DEN, 1%)
Lymphatic endothelial cells (LYME, 1%)
b
Relative gene expression level per cluster
Crabp1
Des
Rgs5
C1qb
Pf4
Eln
Ogn
Cd93
Pecam1
Birc5
Ccnb2
Icos
Nkg7
Ccr7
H2-DMb1
Hdc
G0s2
Cadm4
Itih5
Hba-a2
Hbb-bs
Acp5
Mmp9
Ccl21a
Lyve1
c
Fibroblast heterogeneity
UMAP - 1
UMAP - 2
FIB-A (3%)
FIB-B (14%)
FIB-C (3%)
FIB-D (9%)
FIB-E (4%)
FIB-F (36%)
FIB-G (6%)
FIB-H (20%)
FIB-I (5%)
d
Relative gene expression level per cluster
e
Myeloid heterogeneity
UMAP - 1
UMAP - 2
MYL-A (34%)
MYL-B (38%)
MYL-C (2%)
MYL-D (24%)
MYL-E (3%)
f
Relative gene expression level per cluster
Col1a1
Col1a2
Meg3
Col3a1
Sparc
Apoe
Pf4
Sepp1
Ctsd
Syngr1
2810417H13Rik
Birc5
Top2a
Stmn1
Tuba1b
Cd74
H2-Ab1
Il1b
H2-Aa
H2-Eb1
Col4a1
Col4a2
Igfbp7
Col18a1
Rgs5
g
Endothelial heterogeneity
UMAP - 1
UMAP - 2
ENDO-A (19%)
ENDO-B (8%)
ENDO-C (8%)
ENDO-D (15%)
ENDO-E (43%)
ENDO-F (6%)
h
Relative gene expression level per cluster

Supplementary Figure 2: Classification of skin wound cells into groups. (a) Classification of day 12 skin wound cells into major cell populations. Proportion of cells in each cell group is indicated. **(b)** High-density bar chart showing the distribution of selected marker genes associated with each cell group. Colors correspond to cell populations defined in panel **(a)**. **(c)** Classification of all skin wound fibroblasts into subpopulations. **(d)** Distribution of selected marker genes associated with each fibroblast subpopulation. **(e)** Classification of all skin wound myeloid cells into subpopulations. **(f)** Distribution of selected marker genes associated with each myeloid cell subpopulation. **(g)** Classification of all skin wound endothelial cells into subpopulations. **(h)** The distribution of selected marker genes associated with each endothelial subpopulation.

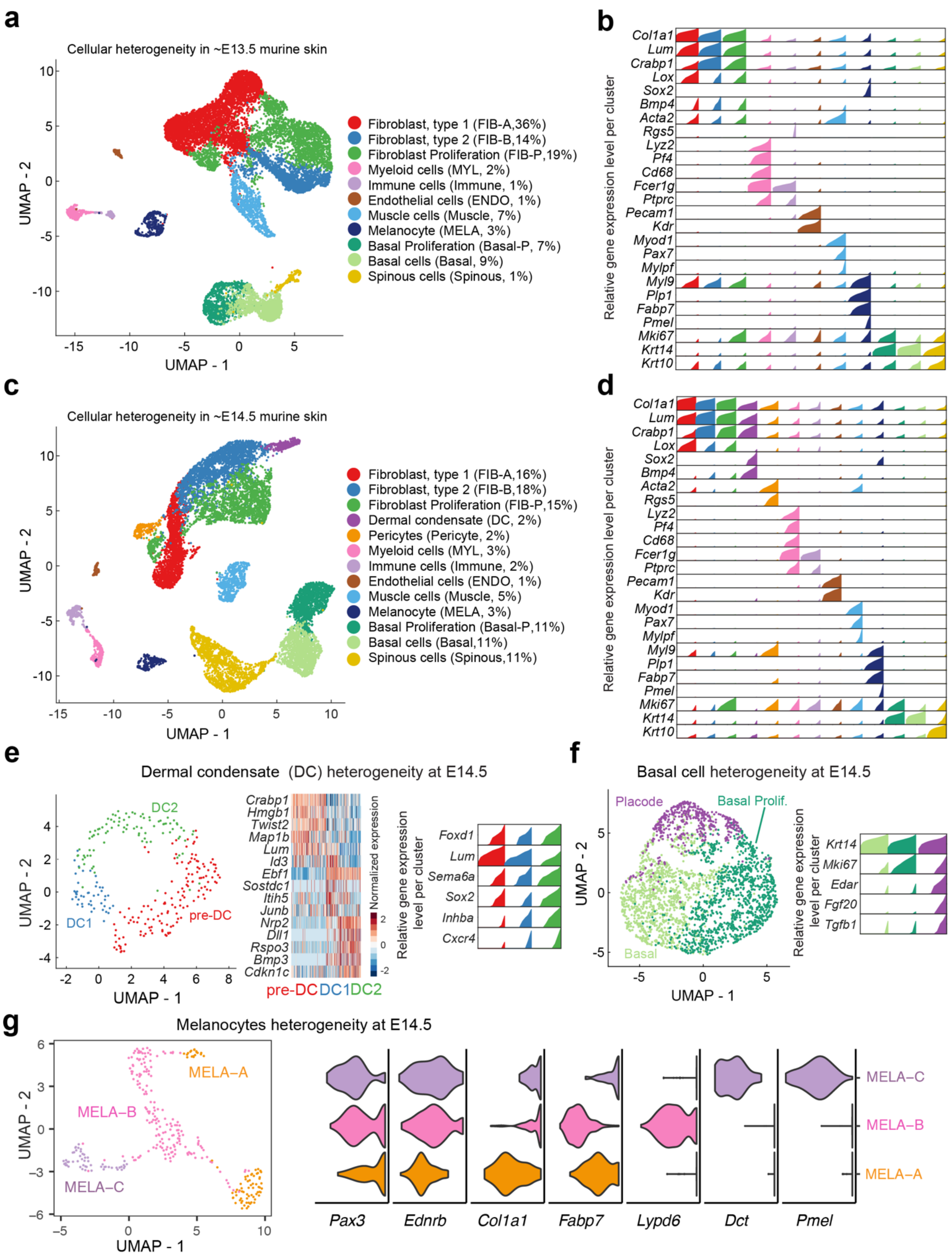
a
Cellular heterogeneity in ~E13.5 murine skin
UMAP - 1
UMAP - 2
Fibroblast, type 1 (FIB-A,36%)
Fibroblast, type 2 (FIB-B,14%)
Fibroblast Proliferation (FIB-P,19%)
Myeloid cells (MYL, 2%)
Immune cells (Immune, 1%)
Endothelial cells (ENDO, 1%)
Muscle cells (Muscle, 7%)
Melanocyte (MELA, 3%)
Basal Proliferation (Basal-P, 7%)
Basal cells (Basal, 9%)
Spinous cells (Spinous, 1%)
b
Relative gene expression level per cluster
Col1a1
Lum
Crabp1
Lox
Sox2
Bmp4
Acta2
Rgs5
Lyz2
Pf4
Cd68
Fcer1g
Ptprc
Pecam1
Kdr
Myod1
Pax7
Mylpf
Myl9
Plp1
Fabp7
Pmel
Mki67
Krt14
Krt10
c
Cellular heterogeneity in ~E14.5 murine skin
UMAP - 1
UMAP - 2
Fibroblast, type 1 (FIB-A,16%)
Fibroblast, type 2 (FIB-B,18%)
Fibroblast Proliferation (FIB-P,15%)
Dermal condensate (DC, 2%)
Pericytes (Pericyte, 2%)
Myeloid cells (MYL, 3%)
Immune cells (Immune, 2%)
Endothelial cells (ENDO, 1%)
Muscle cells (Muscle, 5%)
Melanocyte (MELA, 3%)
Basal Proliferation (Basal-P,11%)
Basal cells (Basal,11%)
Spinous cells (Spinous,11%)
d
Relative gene expression level per cluster
e
Dermal condensate (DC) heterogeneity at E14.5
DC2
DC1
pre-DC
UMAP - 1
UMAP - 2
Crabp1
Hmgb1
Twist2
Map1b
Lum
Id3
Ebf1
Sostdc1
Itih5
Junb
Nrp2
Dll1
Rspo3
Bmp3
Cdkn1c
Normalized expression
pre-DC DC1 DC2
Relative gene expression level per cluster
Foxd1
Lum
Sema6a
Sox2
Inhba
Cxcr4
f
Basal cell heterogeneity at E14.5
Placode
Basal Prolif.
Basal
UMAP - 1
UMAP - 2
Relative gene expression level per cluster
Krt14
Mki67
Edar
Fgf20
Tgfb1
g
Melanocytes heterogeneity at E14.5
MELA–A
MELA–B
MELA–C
UMAP - 1
UMAP - 2
Pax3
Ednrb
Col1a1
Fabp7
Lypd6
Dct
Pmel

Supplementary Figure 3: Classification of cells from embryonic skin dataset into groups. (a) Classification of cells from embryonic E13.5 mouse skin into major cell populations. The proportion of cells in each population is indicated. **(b)** High-density bar chart showing the distribution of selected marker genes associated with each cell population from E13.5. **(c)** Classification of skin cells from embryonic day E14.5 into major cell populations. Proportion of cells in each population is indicated. **(d)** High-density bar chart showing the distribution of selected marker genes associated with each cell population from E14.5. Colors correspond to the cell populations. **(e)** Classification of dermal condensate cells into subpopulations. The heatmap and the distribution of selected marker genes associated with each subpopulation are shown. **(f)** Left: Classification of basal epidermal cells into subpopulations. Right: The distribution of selected marker genes associated with each subpopulation. **(g)** Classification of melanocyte cells into subpopulations. Left: Cells are visualized in the UMAP space and colored by the inferred subpopulations. Right: Violin plot showing the distribution of selected marker genes associated with each subpopulation.

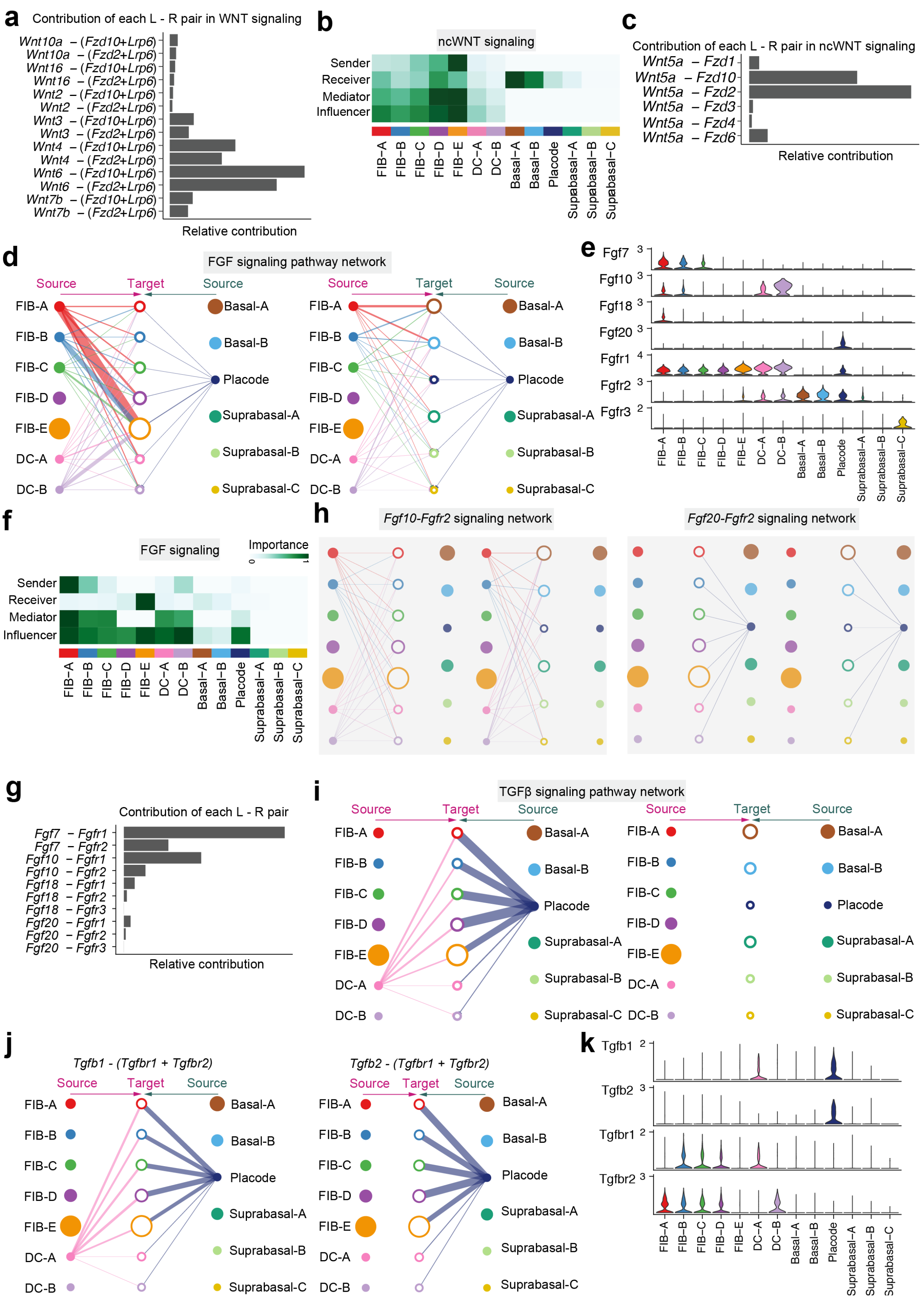
a
Contribution of each L - R pair in WNT signaling
Wnt10a – (Fzd10+Lrp6)
Wnt10a – (Fzd2+Lrp6)
Wnt16 – (Fzd10+Lrp6)
Wnt16 – (Fzd2+Lrp6)
Wnt2 – (Fzd10+Lrp6)
Wnt2 – (Fzd2+Lrp6)
Wnt3 – (Fzd10+Lrp6)
Wnt3 – (Fzd2+Lrp6)
Wnt4 – (Fzd10+Lrp6)
Wnt4 – (Fzd2+Lrp6)
Wnt6 – (Fzd10+Lrp6)
Wnt6 – (Fzd2+Lrp6)
Wnt7b – (Fzd10+Lrp6)
Wnt7b – (Fzd2+Lrp6)
Relative contribution
b
ncWNT signaling
Sender
Receiver
Mediator
Influencer
FIB–A
FIB–B
FIB–C
FIB–D
FIB–E
DC–A
DC–B
Basal–A
Basal–B
Placode
Suprabasal–A
Suprabasal–B
Suprabasal–C
c
Contribution of each L - R pair in ncWNT signaling
Wnt5a – Fzd1
Wnt5a – Fzd10
Wnt5a – Fzd2
Wnt5a – Fzd3
Wnt5a – Fzd4
Wnt5a – Fzd6
Relative contribution
d
FGF signaling pathway network
Source
Target
Source
FIB-A
FIB-B
FIB-C
FIB-D
FIB-E
DC-A
DC-B
Basal-A
Basal-B
Placode
Suprabasal-A
Suprabasal-B
Suprabasal-C
e
Fgf7
Fgf10
Fgf18
Fgf20
Fgfr1
Fgfr2
Fgfr3
f
FGF signaling
Importance
Sender
Receiver
Mediator
Influencer
h
Fgf10-Fgfr2 signaling network
Fgf20-Fgfr2 signaling network
g
Contribution of each L - R pair
Fgf7 – Fgfr1
Fgf7 – Fgfr2
Fgf10 – Fgfr1
Fgf10 – Fgfr2
Fgf18 – Fgfr1
Fgf18 – Fgfr2
Fgf18 – Fgfr3
Fgf20 – Fgfr1
Fgf20 – Fgfr2
Fgf20 – Fgfr3
Relative contribution
i
TGFβ signaling pathway network
j
Tgfb1 - (Tgfbr1 + Tgfbr2)
Tgfb2 - (Tgfbr1 + Tgfbr2)
k
Tgfb1
Tgfb2
Tgfbr1
Tgfbr2

Supplementary Figure 4: Example of intercellular communication networks for continuous cell states along cell pseudotemporal trajectories. (a) Relative contribution of each ligand-receptor pair to the overall communication network of WNT signaling pathway, which is the ratio of the total communication probability of the inferred network of each ligand-receptor pair to that of a signaling pathway. **(b)** Heatmap showing the relative importance of each cell group based on the computed four network centrality measures of ncWNT signaling. **(c)** Relative contribution of each ligand-receptor pair to the overall communication network of ncWNT signaling pathway. **(d)** Hierarchical plot showing dermal and epidermal communications via FGF signaling. The hierarchical plot consists of two parts: Left and right portions highlight the autocrine and paracrine signaling to dermal and epidermal populations, respectively. Solid and open circles represent source and target, respectively. Circle sizes are proportional to the number of cells in each cell group and edge width represents the communication probability. Edge colors are consistent with the signaling source. **(e)** Violin plot showing the expression patterns of signaling genes involved in the inferred FGF signaling network. Normalized expression levels are shown in the violin plot. **(f)** Heatmap showing the relative importance of each cell group based on the computed four network centrality measures of FGF signaling. **(g)** Relative contribution of each ligand-receptor pair to the overall communication network of FGF signaling pathway. **(h)** Inferred intercellular communication networks of two ligand-receptor pairs, Fgf10-Fgfr2 and Fgf20-Fgfr2. **(i)** Inferred intercellular communication networks of TGFβ signaling pathway. **(j)** Inferred intercellular communication networks of two ligand-receptor pairs, *Tgfb1 – (Tgfbr1+Tgfbr2)* and *Tgfb2 – (Tgfbr1+Tgfbr2).* **(k)** Violin plot showing the expression patterns of signaling genes involved in the inferred TGFb signaling network.

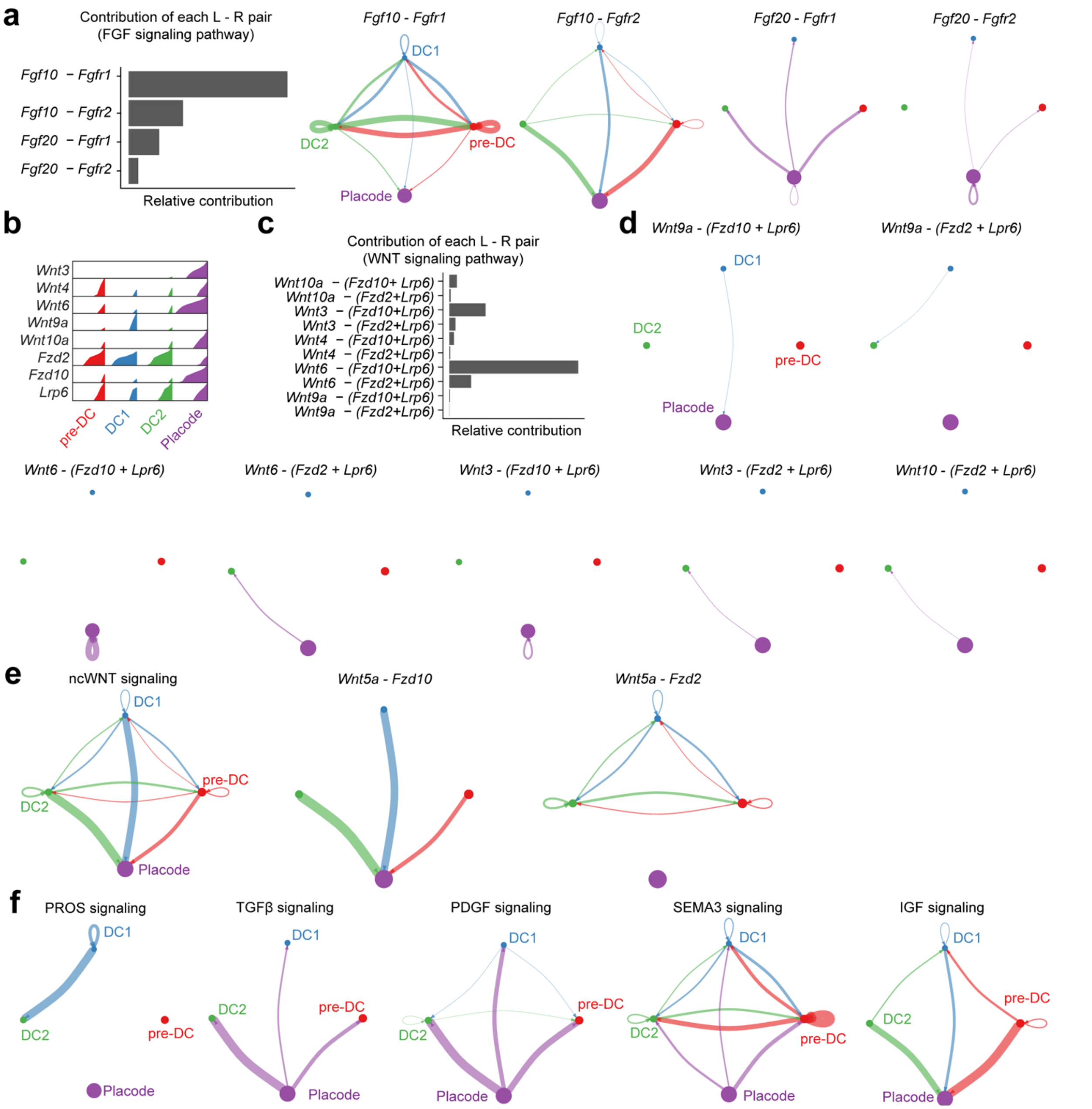

Supplementary Figure 5: Example of intercellular communication networks for spatially colocalized DC and placode cell populations. (a) Left: Relative contribution of each ligand-receptor pair to the overall communication network of FGF signaling pathway, which is the ratio of the total communication probability of the inferred network of each ligand-receptor pair to that of FGF signaling pathway. Right: Inferred intercellular communication network for each ligand-receptor pair associated with FGF signaling pathway. Circle sizes are proportional to the number of cells in each cell group and edge width represents the communication probability. **(b)** The distribution of several WNT pathway signaling genes in each cell group. **(c)** Left: Relative contribution of each ligand-receptor pair to the overall communication network of WNT

signaling pathway. Right: Inferred intercellular communication network for some ligand-receptor pairs associated with WNT signaling pathway. **(d)** Inferred intercellular communication network of WNT signaling pathway and associated ligand-receptor pairs. **(e)** Inferred intercellular communication network of ncWNT signaling pathway and associated ligand-receptor pairs. **(f)** Inferred intercellular communication networks for PROS, TGFβ, PDGF, SEMA3 and IGF signaling pathways.

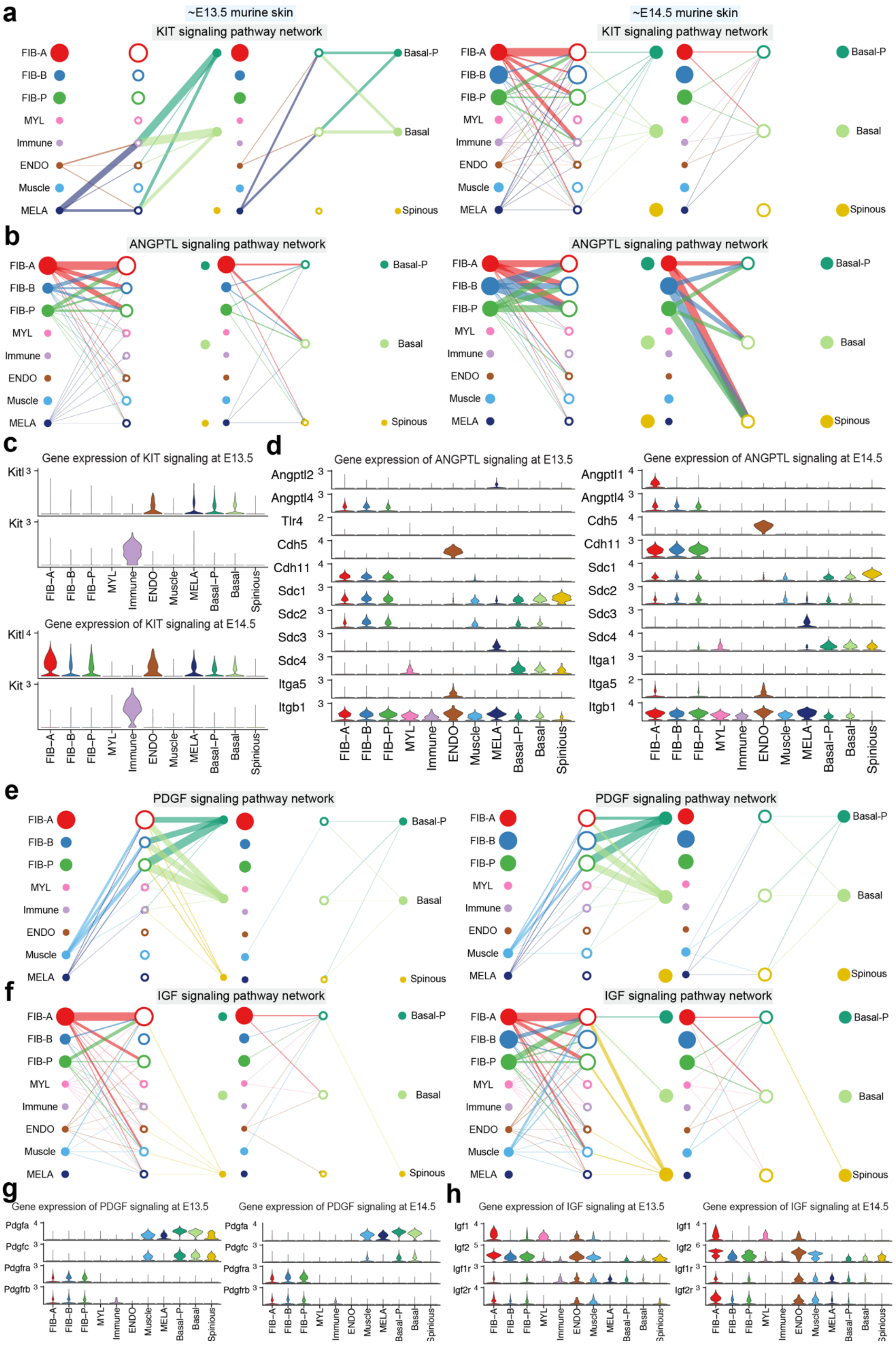
a
~E13.5 murine skin
KIT signaling pathway network
~E14.5 murine skin
KIT signaling pathway network
FIB-A
FIB-B
FIB-P
MYL
Immune
ENDO
Muscle
MELA
Basal-P
Basal
Spinous
b
ANGPTL signaling pathway network
ANGPTL signaling pathway network
c
Gene expression of KIT signaling at E13.5
Kitl
Kit
Gene expression of KIT signaling at E14.5
Kitl
Kit
d
Gene expression of ANGPTL signaling at E13.5
Angptl2
Angptl4
Tlr4
Cdh5
Cdh11
Sdc1
Sdc2
Sdc3
Sdc4
Itga5
Itgb1
Gene expression of ANGPTL signaling at E14.5
Angptl1
Angptl4
Cdh5
Cdh11
Sdc1
Sdc2
Sdc3
Sdc4
Itga1
Itga5
Itgb1
e
PDGF signaling pathway network
PDGF signaling pathway network
f
IGF signaling pathway network
IGF signaling pathway network
g
Gene expression of PDGF signaling at E13.5
Gene expression of PDGF signaling at E14.5
Pdgfa
Pdgfc
Pdgfra
Pdgfrb
h
Gene expression of IGF signaling at E13.5
Gene expression of IGF signaling at E14.5
Igf1
Igf2
Igf1r
Igf2r

Supplementary Figure 6: Comparison of example intercellular communication networks between E13.5 and E14.5 embryonic skin. (a, b, e, f) Inferred intercellular communication networks for KIT, ANGPTL, PDGF and IGF signaling pathways at E13.5 and E14.5. The hierarchical plot consists of two parts: Left and right portions highlight the autocrine and paracrine signaling to dermal and epidermal populations, respectively. Solid and open circles represent source and target, respectively. Circle sizes are proportional to the number of cells in each cell group and edge width represents the communication probability. **(c, d, g, h)** Violin plot showing the expression patterns of signaling genes involved in each inferred signaling network at E13.5 and E14.5. Normalized expression levels are used.

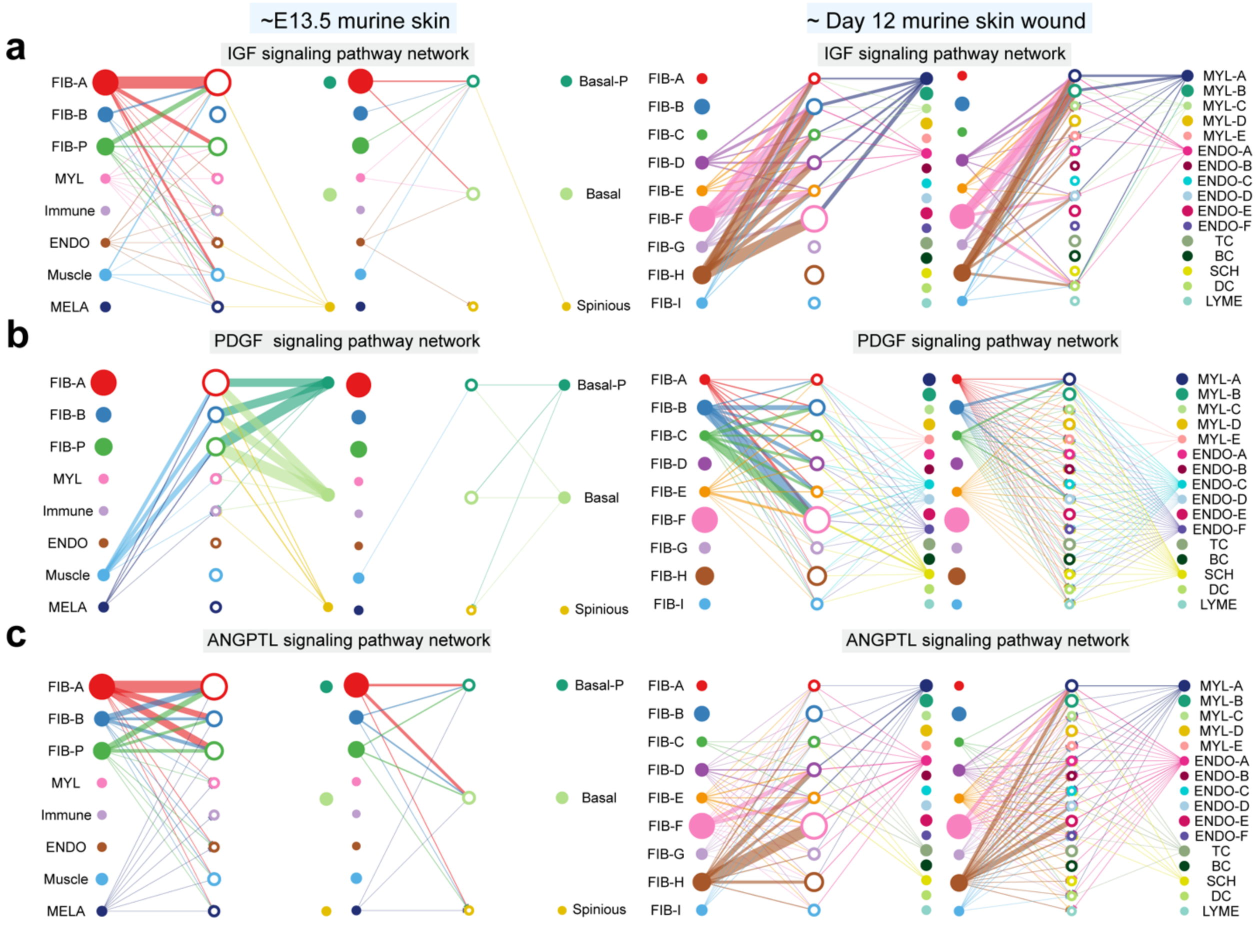

Supplementary Figure 7: Comparison of example intercellular communication networks between E13.5 embryonic skin and adult skin wound. (a-c) Inferred intercellular communication networks for IGF, PDGF and ANGPTL signaling pathways in embryonic skin at E13.5 and adult skin wound at day 12. Solid and open circles represent source and target, respectively. Circle sizes are proportional to the number of cells in each cell group and edge width represents the communication probability.

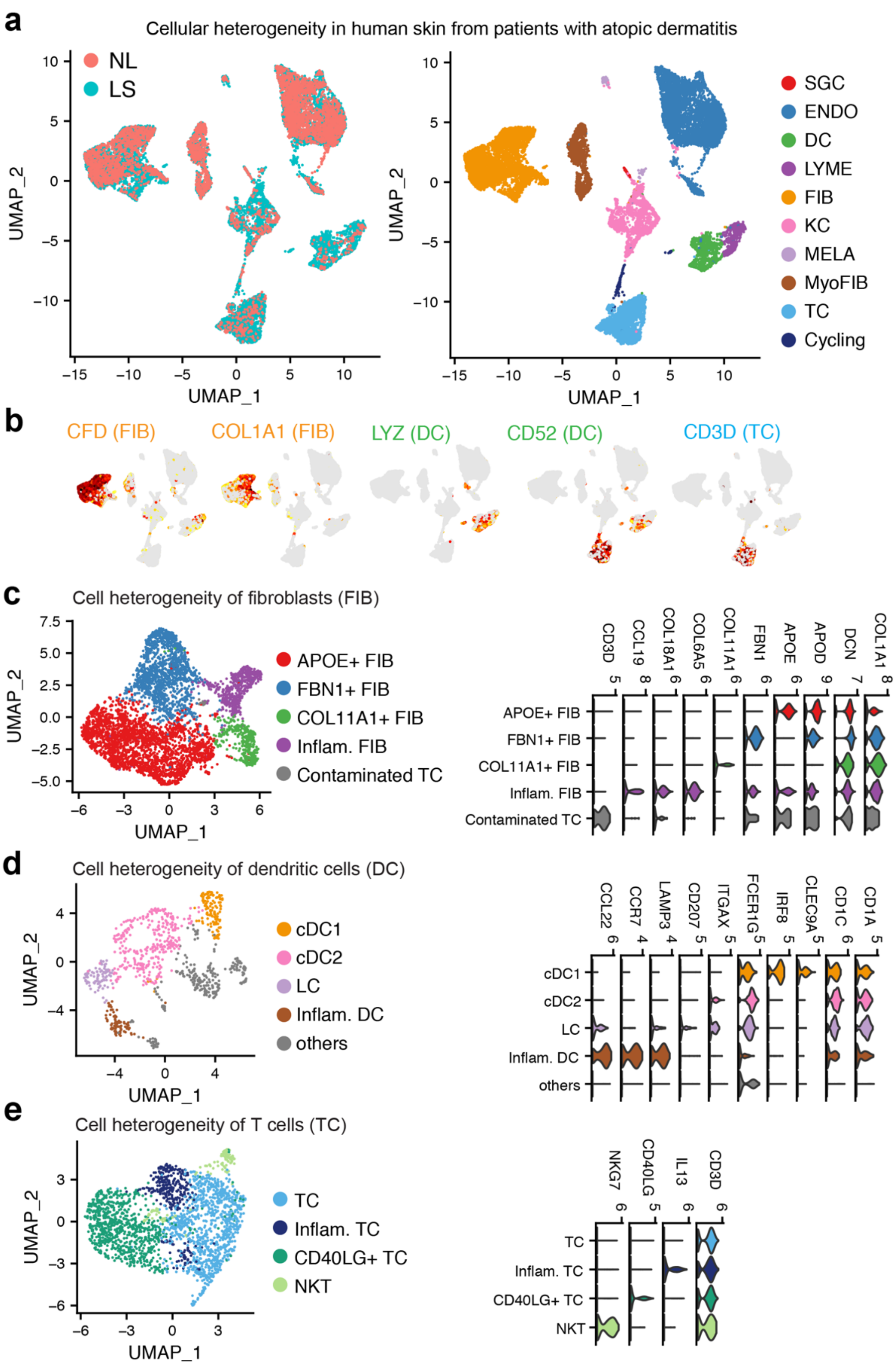
a
Cellular heterogeneity in human skin from patients with atopic dermatitis
NL
LS
SGC
ENDO
DC
LYME
FIB
KC
MELA
MyoFIB
TC
Cycling
UMAP_1
UMAP_2
b
CFD (FIB)
COL1A1 (FIB)
LYZ (DC)
CD52 (DC)
CD3D (TC)
c
Cell heterogeneity of fibroblasts (FIB)
APOE+ FIB
FBN1+ FIB
COL11A1+ FIB
Inflam. FIB
Contaminated TC
CD3D
CCL19
COL18A1
COL6A5
COL11A1
FBN1
APOE
APOD
DCN
COL1A1
d
Cell heterogeneity of dendritic cells (DC)
cDC1
cDC2
LC
Inflam. DC
others
CCL22
CCR7
LAMP3
CD207
ITGAX
FCER1G
IRF8
CLEC9A
CD1C
CD1A
e
Cell heterogeneity of T cells (TC)
TC
Inflam. TC
CD40LG+ TC
NKT
NKG7
CD40LG
IL13
CD3D

Supplementary Figure 8: Classification of cells from human skin dataset into groups. (a) Classification of skin cells from human patients with atopic dermatitis into major cell populations. Cells are colored according to skin biopsy sites and annotated cell types, respectively. SGC: Sweat gland cell; ENDO: endothelial cells; DC: Dendritic cell; LYME: Lymphatic endothelial cell; FIB, fibroblast; KC: keratinocyte; MELA, melanocyte; Myo-FIB: myofibroblast; NKT, natural killer T cell; cycling: cells expressing cell cycle markers. **(b)** Feature plot showing the expression pattern of marker genes associated with FIB, DC and TC. Dark red colors indicate high expression. **(c-e)** Classification of fibroblast, DC and TC into subpopulations independently. The distribution of selected marker genes associated with each subpopulation are shown.

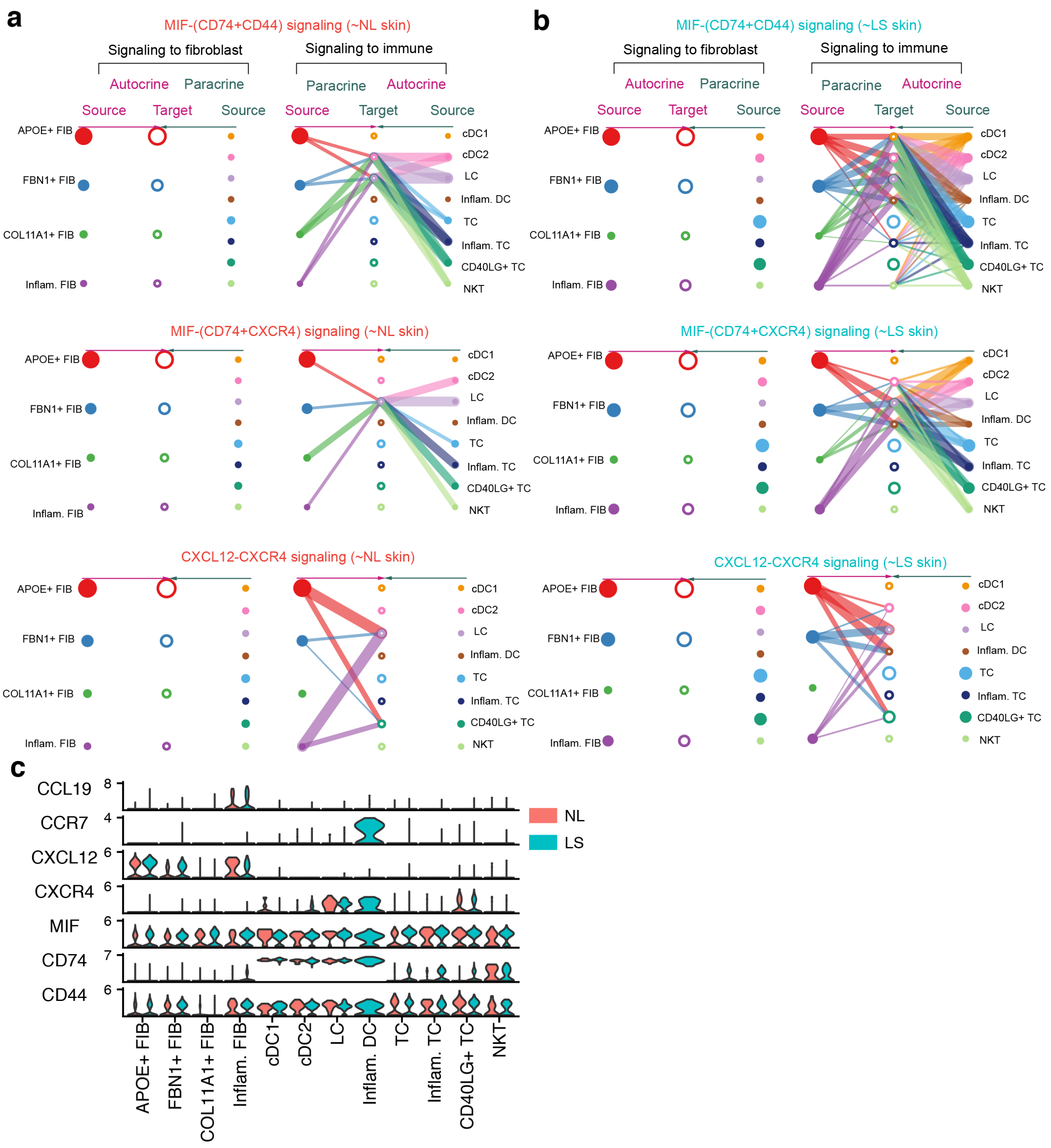

Supplementary Figure 9: Comparison of example intercellular communication networks between nonlesional (NL) and lesional (LS) skin from the human skin dataset. (a-b) Inferred intercellular communication networks for MIF and CXCL signaling pathways from NL and LS skin. The hierarchical plot consists of two parts: Left and right portions highlight the autocrine and paracrine signaling to fibroblast and immune cells, respectively. Solid and open circles represent source and target, respectively. Circle sizes are proportional to the number of cells in each cell group and edge width represents the communication

probability. Edge colors are consistent with the signaling source. **(c)** The expression distribution of related signaling genes are shown. Red and green colors represent the normalized expression levels from NL and LS samples, respectively.

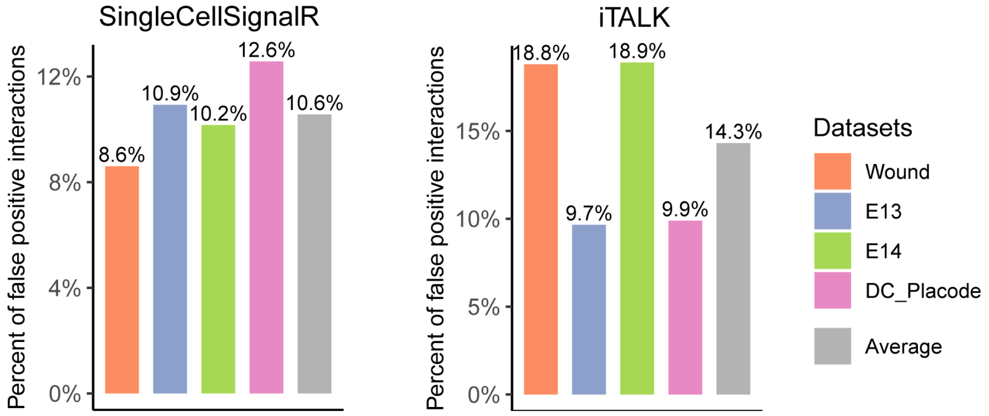

Supplementary Figure 10: The percentage of false positive interactions caused by incomplete representations of known signaling molecule interactions. We compute the percentage of false positive interactions inferred by SingleCellSignalR and iTALK. The false positive interactions are defined by the interactions with multi-subunits that are *partially* identified by these tools. The interactions with multi-subunits are from CellChatDB.

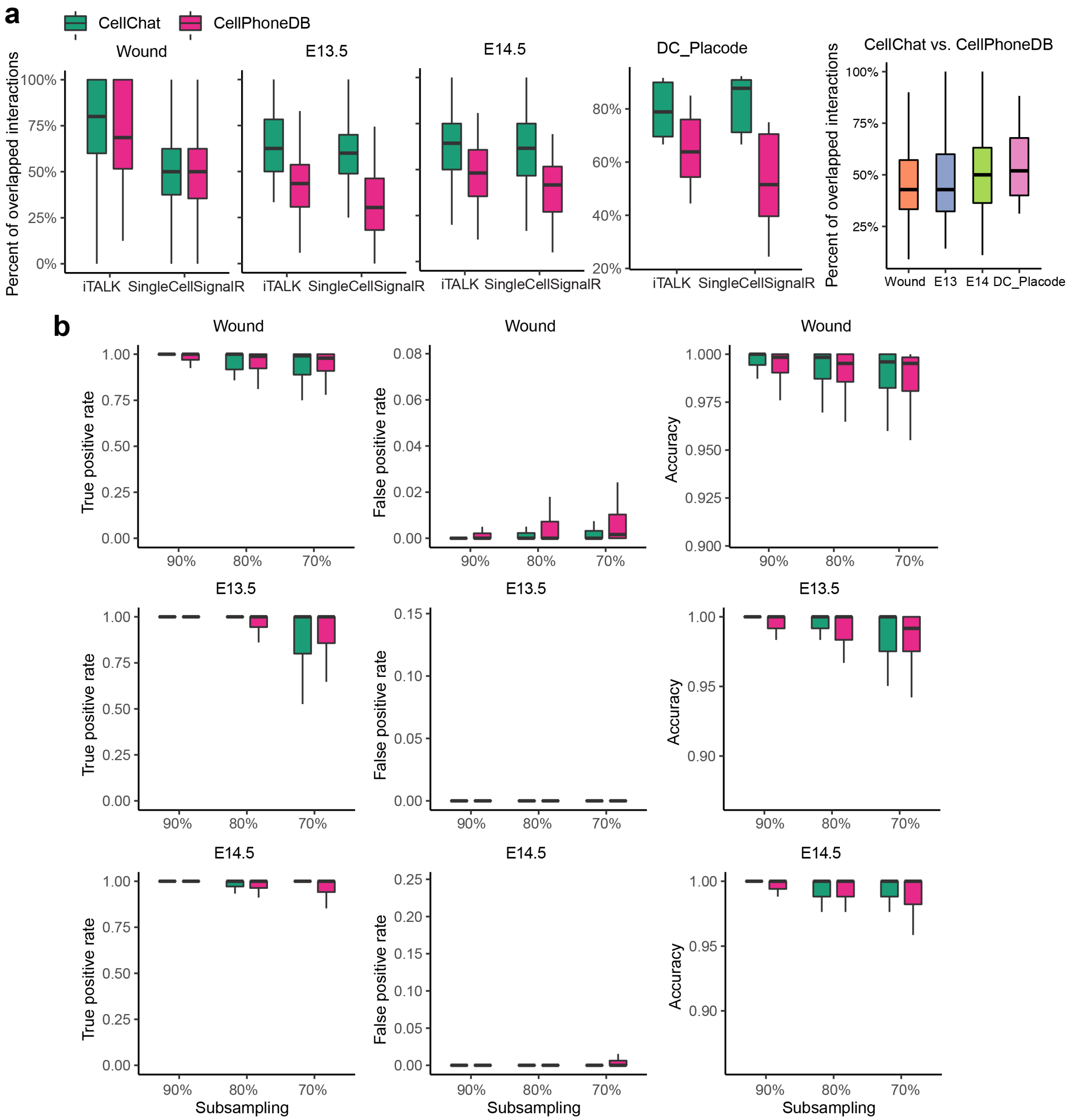

Supplementary Figure 11: Comparison of ligand-receptor interactions predicted by CellChat and CellPhoneDB. (a) The percentages of overlapped ligand-receptor interactions between CellChat/CellPhoneDB and other two methods including SingleCellSignalR and iTALK are shown. The percentage of overlapped ligand-receptor interactions between CellChat and CellPhoneDB is also shown. The sample size used to create a box plot is dependent on the number of cell groups in the dataset. Wound dataset: *n* = 625 biologically independent samples; E13.5 dataset: *n* = 121 biologically independent samples; E14.5 dataset: *n* = 169 biologically independent samples; DC_Placode: *n* = 16 biologically independent

samples. The overlap percentage is defined as the number of overlapped ligand-receptor interactions divided by the minimum number of identified ligand-receptor interactions between two methods. **(b)** Comparison of the robustness of inferred interactions under subsampling (90%, 80%, 70%) of the cells from each dataset. The sample size used to create a box plot is dependent on the number of inferred ligand-receptor pairs in the dataset. Wound dataset: *n* = 60 for CellChat and *n* = 273 for CellPhoneDB biologically independent samples; E13.5 dataset: *n* = 89 for CellChat and *n* = 359 for CellPhoneDB biologically independent samples; E14.5 dataset: *n* = 109 for CellChat and *n* = 455 for CellPhoneDB biologically independent samples. In each case, we compute three measures including true positive rate (TPR), false positive rate (FPR) and accuracy (ACC) by comparing the inferred interactions from subsampled dataset with those from the original dataset. In each box-plot, the black center line indicate median value, and the thin black lines extend to the most extreme values within 1.5 times the IQR (interquartile range) of the median.

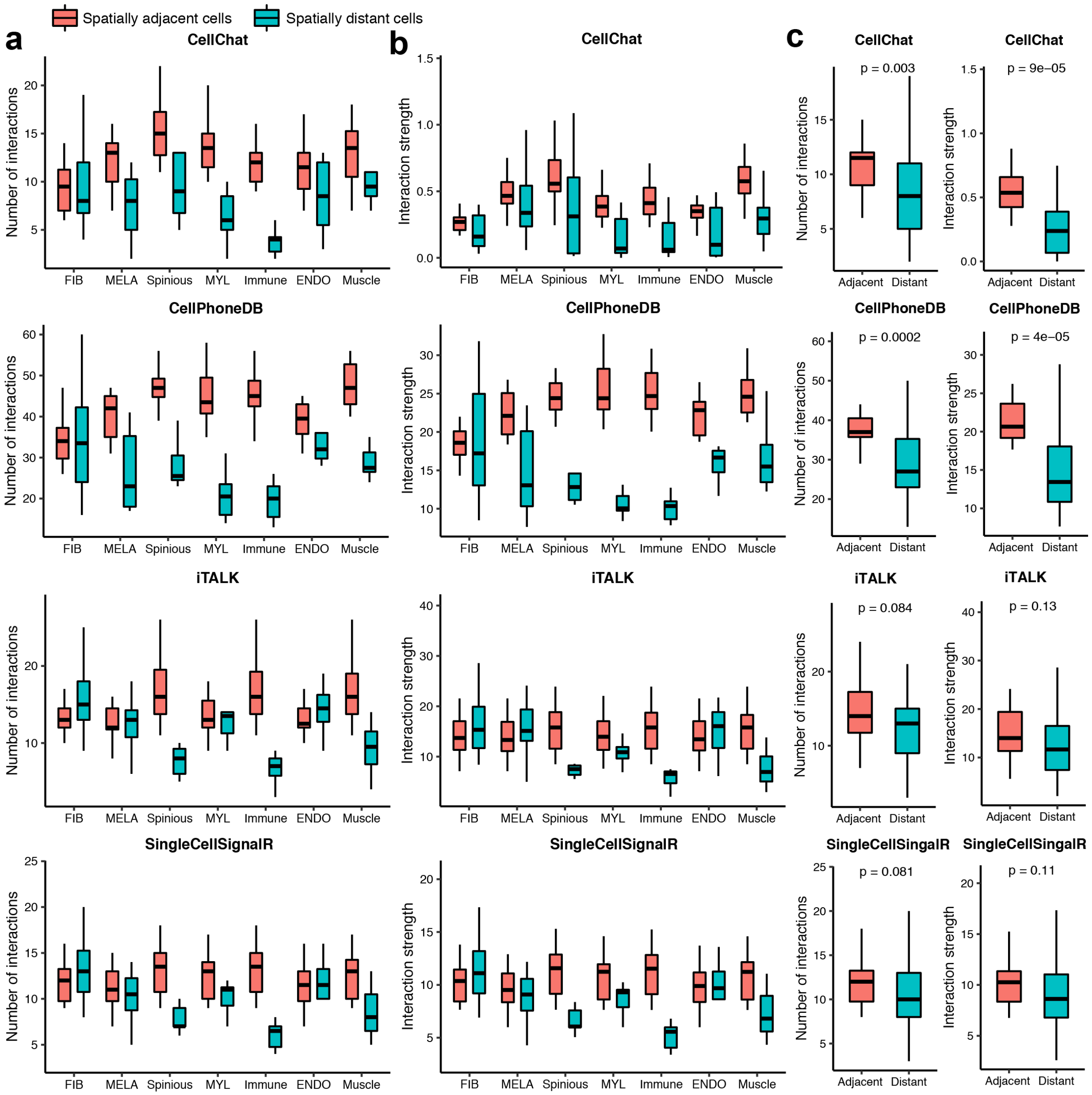

Supplementary Figure 12: Performance of different computational methods in distinguishing the spatially adjacent from distant cells. (a) Comparison of the number of inferred interactions between spatially adjacent and distant cells for each method. The interactions among spatially adjacent cells are the interactions among the four spatially colocalized cell populations, including placode, preDC, DC1 and DC2 subpopulations. The interactions among spatially distant cells are the interactions between each cell type indicated on the x-axis and the four spatially colocalized cell populations. Note that here we only retained the top 10% of L-R pairs (the most significant) inferred by iTALK and SingleCellSignalR to ensure the comparable number of L-R pairs with that by CellChat. We considered seven cell types from E14.5 embryonic skin dataset that are likely not spatially adjacent to the four spatially colocalized cell populations,

including FIB (fibroblasts), MELA (melanocytes), Spinous (spinous epithelial cells), MYL (myeloid cells), Immune (other immune cells), ENDO (endothelial cells) and Muscle. The sample size used to create a box plot is dependent on the number of cell groups in each cell type. FIB: *n* = 12 biologically independent samples for red colored box plot and *n* = 24 for green colored box plot; MELA: *n* = 12 biologically independent samples for red colored box plot and *n* = 16 for green colored box plot; Spinous, MYL, Immune, ENDO and Muscle: *n* = 12 biologically independent samples for red colored box plot and *n* = 8 for green colored box plot. **(b)** Comparison of the interaction strength between spatially adjacent and distant cells. The interaction strength is calculated by the interaction probability or score. The sample size in each box plot is the same as panel (a). **(c)** Comparison of the overall number of inferred interaction as well as the overall interaction strength by considering all spatially distant cells together, i.e., considering all the seven cell types together indicated in panel (a). Red and green colored boxes correspond to *n* = 12 and 88 biologically independent samples respectively. p-values are from two-sided Wilcoxon rank-sum tests. Both CellChat and CellPhoneDB performed well at discriminating spatially adjacent from distant cells. In each box-plot, the black center line indicate median value, and the thin black lines extend to the most extreme values within 1.5 times the IQR (interquartile range) of the median.

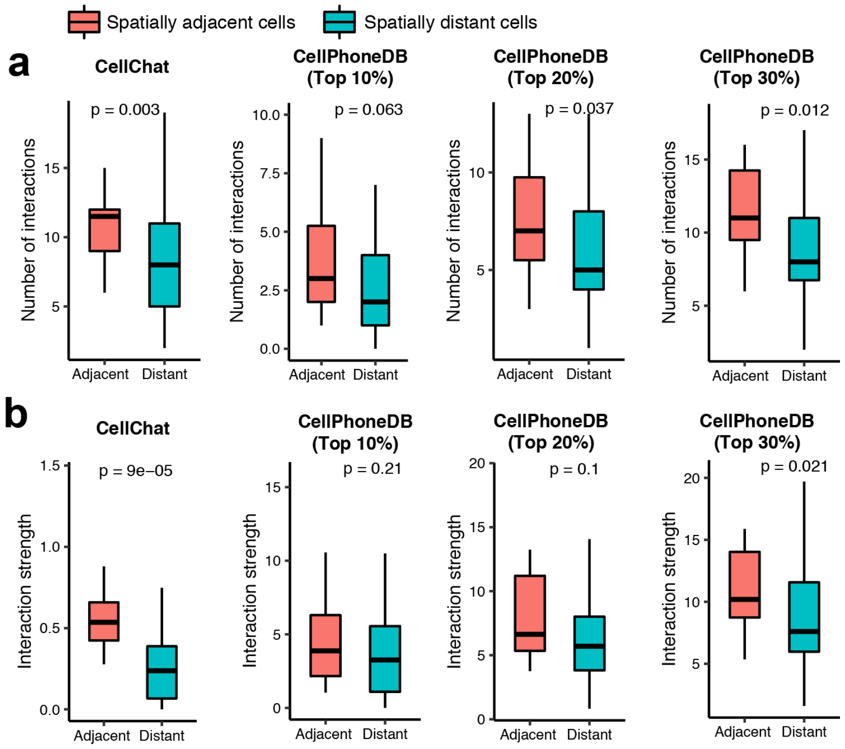

Supplementary Figure 13: Performance of the top interactions predicted by CellPhoneDB in distinguishing the spatially adjacent from distant cells. (a) Comparison of the number of inferred interactions between spatially adjacent and distant cells for the top 10%, top 20% and top 30% interactions predicted by CellPhoneDB. The top interactions are the strongest interactions that are measured by the interaction scores in CellPhoneDB. The results from CellChat was presented here as a comparison. The definitions of spatially adjacent and distant cells can be found in the legend in Supplementary Figure 12. Red and green colored boxes correspond to n = 12 and 88 biologically independent samples respectively. **(b)** Comparison of the number of the interaction strength between spatially adjacent and distant cells for the top 10%, top 20% and top 30% interactions predicted by CellPhoneDB. In each box-plot, the black center line indicate median value, and the thin black lines extend to the most extreme values within 1.5 times the IQR (interquartile range) of the median. Red and green colored boxes correspond to n = 12 and 88 biologically independent samples respectively. p-values are from two-sided Wilcoxon rank-sum tests. The difference between spatially adjacent and spatially distant cells predicted by CellPhoneDB here was not as significant as with that predicted by CellChat, suggesting that CellChat performed better at capturing stronger interactions.

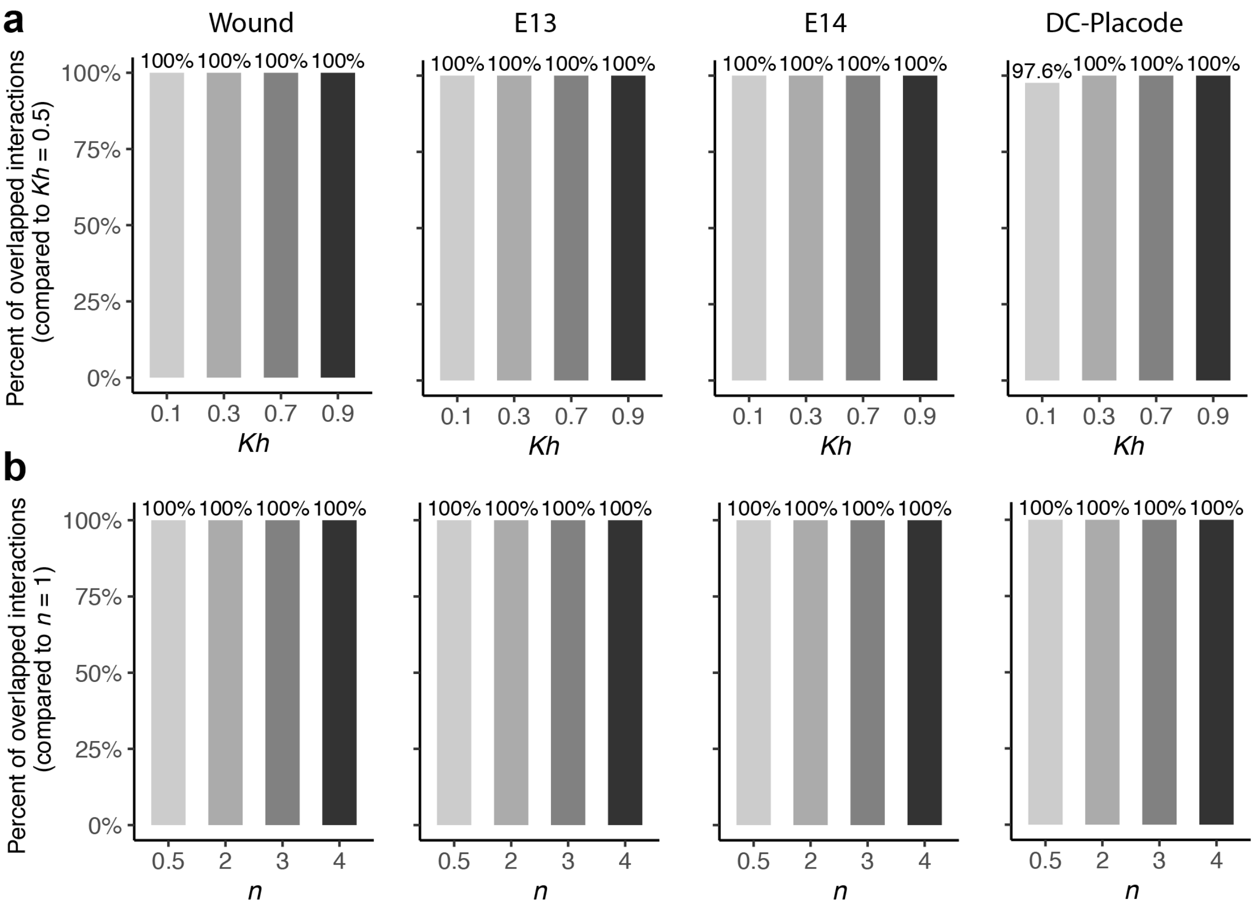

Supplementary Figure 14: Robustness of the inferred interactions by CellChat with respect to different parameters in the Hill function. (a) The overlapped interactions between *Kh* = 0.5 (default) and *Kh* = 0.1, 0.3, 0.7, 0.9 for each dataset. **(b)** The overlapped interactions between *n* = 1 (default Hill coefficient) and *n* = 0.5, 2, 3, 4 for each dataset. We varied the dissociation constant *Kh* from 0.1 to 0.9 with an increment of 0.2, and then computed the Jaccard similarity between the interactions inferred with each varied *Kh* and the interactions inferred with *Kh* being 0.5. We found the inference is relatively robust to the choice of *Kh* for all the four tested datasets. Similarly, by varying Hill coefficient *n* from 0.5 to 4, we also found the inferred ligand-receptor interactions are relatively robust.

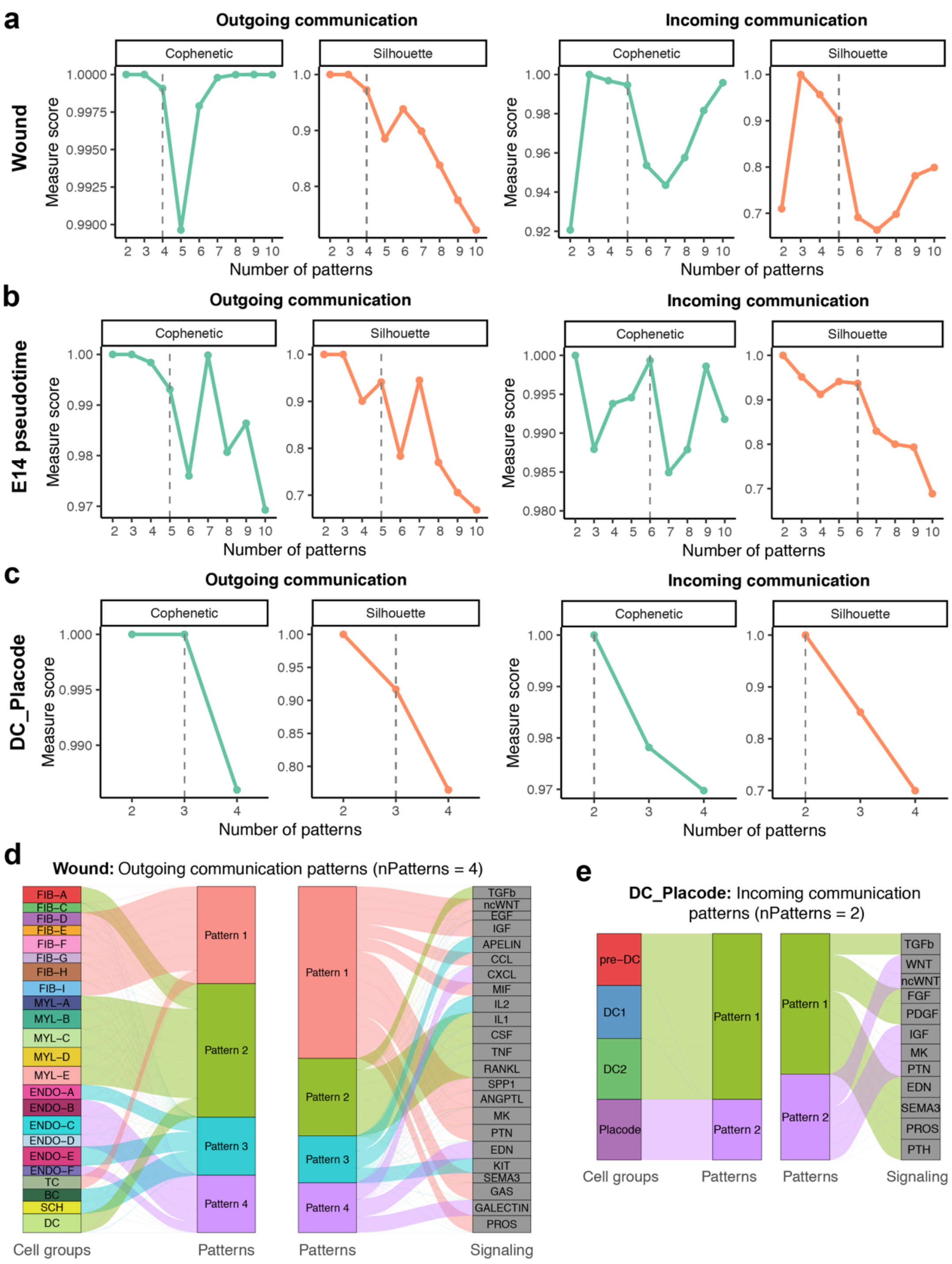

Supplementary Figure 15: The number of patterns inferred by the Cophenetic and Silhouette metrics. (a-c) For the outgoing and incoming communication analyses in each studied dataset, the computed Cophenetic and Silhouette scores are shown for a predefined range of the number of patterns. The dashed lines indicate a suitable number of patterns. A suitable number of patterns is the one at which Cophenetic and Silhouette values begin to drop suddenly. (**d**) The alluvial plot showing outgoing signaling patterns of

secreting cells in the wound dataset based on the number of patterns being 4, which shows the correspondence between the inferred latent patterns and cell groups as well as signaling pathways. The thickness of the flow indicates the contribution of the cell group or signaling pathway to each latent pattern. The height of each pattern is proportional to the number of its associated cell groups or signaling pathways. Outgoing patterns reveal how the sender cells coordinate with each other as well as how they coordinate with certain signaling pathways to drive signals. **(e**) The alluvial plot showing incoming signaling patterns of target cells in the DC_Placode dataset based on the number of patterns equal to 2. Incoming patterns show how the target cells coordinate with each other as well as how they coordinate with certain signaling pathways to respond to signals.

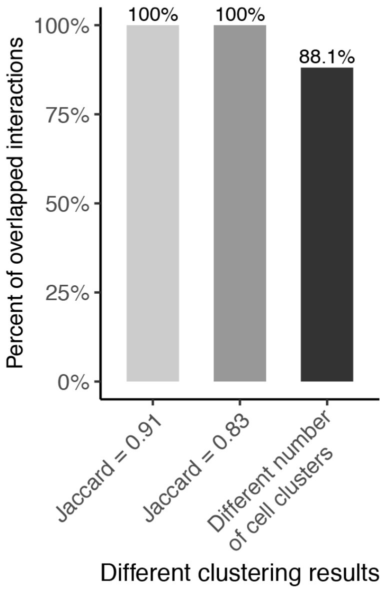

Supplementary Figure 16: Robustness of the inferred interactions by CellChat with respect to different clustering results. The overlapped interactions between the original clustering results and another three different clustering results using the four spatially colocalized cell subpopulations (i.e., placode, preDC, DC1 and DC2) as an example. We used two different choices of parameters to produce two different clustering results while keeping the number of cell clusters unchanged. The Jaccard similarities between these two new clustering results and our original clustering result are 0.91 and 0.83, respectively. In addition, we also used another choice of parameters to produce different number of cell clusters (three subpopulations: placode, preDC, DC).

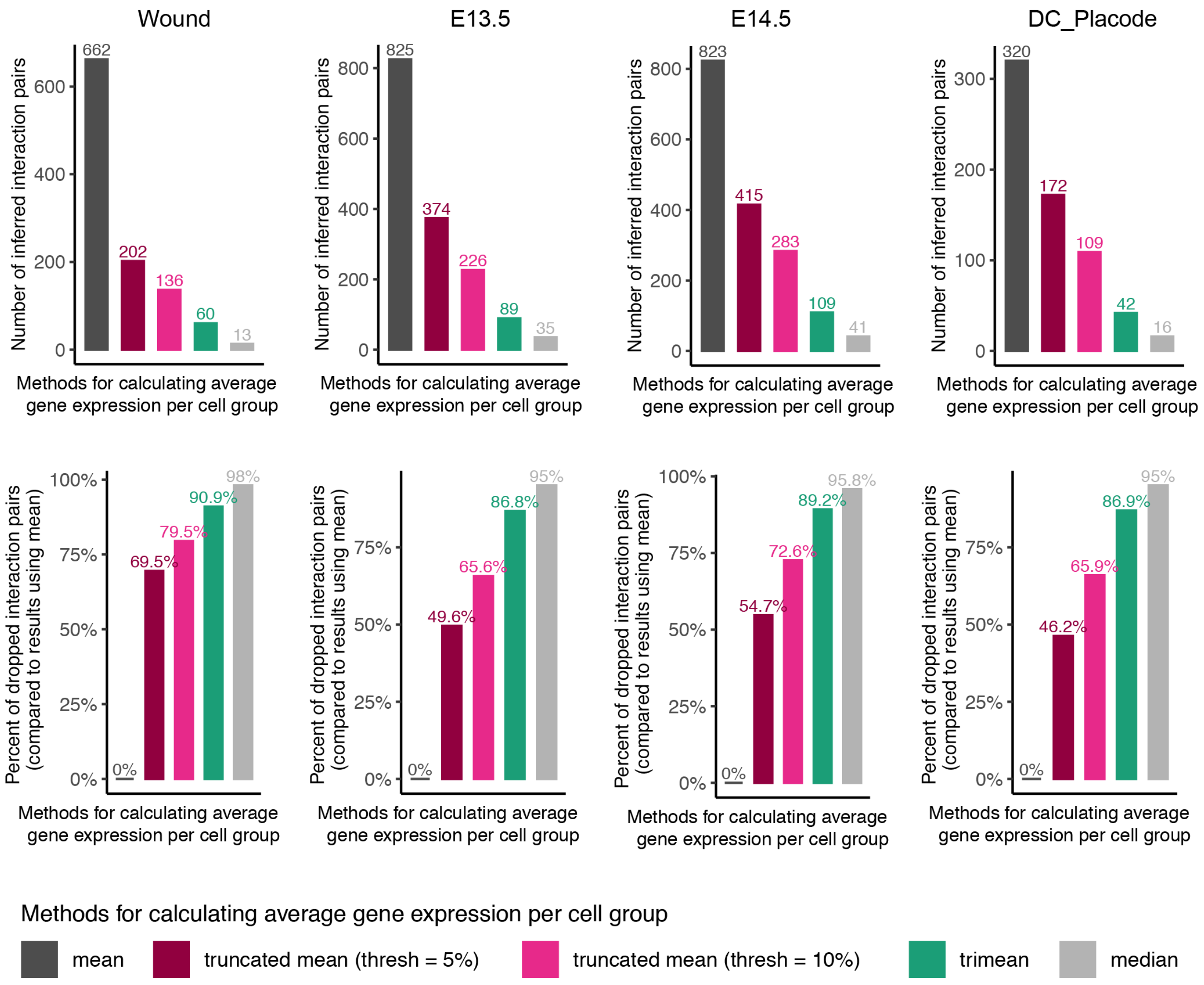

Supplementary Figure 17: The number of inferred ligand-receptor pairs using different methods for calculating the average gene expression per cell group. For each studied dataset, the number of inferred ligand-receptor pairs (upper panel) and the percent of dropped pairs (lower panel) are computed using one of the five methods for calculating the average gene expression per cell group. The percent of dropped pairs is computed using the result from the 'mean' method as a baseline. The five methods include mean (i.e., simply calculating the average gene expression), 5% truncated mean (i.e., calculating the average gene expression by discarding 5% from each end of the data), 10% truncated mean, trimean (i.e., the method used in CellChat) and median.

Supplementary Table

Supplementary Table 1. Comparison of the inferred intercellular communication networks among the four methods including CellChat, CellPhoneDB, iTALK and SingleCellSignalR. For any two cell subpopulations (each line in the table), we calculated the number of L-R pairs inferred by each method, and the number of shared L-R pairs between any two methods, using an example of four spatially colocalized cell populations in E14.5 embryonic mouse skin. Note that here we only retained the top 10% of L-R pairs (the most significant) inferred by iTALK and SingleCellSignalR to ensure the comparable number of L-R pairs with that by CellChat. SingleCellSignalR shared very few L-R pairs with other three methods, suggesting a very different logic used by SingleCellSignalR for quantifying and ranking L-R interactions. In the table, SignalR represents SingleCellSignalR.

	Number of inferred LR interactions				Number of shared LR interactions					
	CellChat	CellPhoneDB	iTALK	SignalR	CellChat & CellPhoneDB	CellChat &iTALK	CellChat &SignalR	CellPhoneDB & iTALK	CellPhoneDB & SignalR	iTALK &SignalR
pre-DC -> pre-DC	12	29	11	11	9	4	0	4	1	0
pre-DC -> DC1	11	37	14	12	9	3	0	3	2	1
pre-DC -> DC2	10	36	17	13	6	5	0	3	1	1
pre-DC -> Placode	8	38	9	8	5	1	0	2	0	0
DC1 -> pre-DC	13	30	12	12	6	4	0	3	0	0
DC1 -> DC1	9	35	19	13	7	4	0	5	1	1
DC1 -> DC2	12	32	18	16	5	4	0	4	1	1
DC1 -> Placode	6	40	11	9	5	2	0	2	0	0
DC2 -> pre-DC	13	36	15	10	8	5	0	7	0	0
DC2 -> DC1	9	40	23	13	8	5	0	8	1	3
DC2 -> DC2	11	37	24	18	6	7	0	9	1	3
DC2 -> Placode	7	42	13	9	5	1	0	2	0	0
Placode -> pre-DC	15	36	12	11	7	6	0	4	0	1
Placode -> DC1	12	44	14	14	5	5	0	4	1	2
Placode -> DC2	18	44	15	14	6	7	0	6	0	2
Placode -> Placode	13	50	7	8	11	2	0	3	1	0

Supplementary Table 2. Characterization and comparison of CellChat with other tools for intercellular communication analysis. Note: SingleCellSignalR only provides a ligand-receptor database LRdb in human, but it internally maps mouse genes to their human orthologs according to Ensembl.

		CellChat	SingleCellSignalR	iTALK	CellPhoneDB
**Database**	Multisubunit structure	Y	N	N	Y
	Cofactors	Y	N	N	N
	Structured pathways	Y	N	N	N
	Number of interactions	2021/1939	3251	2648	1396
	Species	mouse/human	human	human	human
**Input data**	Preprocessed data matrix	Y	Y	Y	Y
	Low-dimensional space	Y	N	N	N
	Multiple datasets	Y	N	Y	N
**Model**	Methodology	Mass Action Law + Statistical test	Regularized product + Thresholding	DEG analysis	Mean expression + Statistical test
	Cell proportion	Y	N	N	N
**Systems analysis**	Infer signaling roles of cells	Y	N	N	N
	Predict key incoming and outgoing signals	Y	N	N	N
	Predict signaling patterns	Y	N	N	N
	Classify signaling networks	Y	N	N	N
	Identify conserved vs. context-specific signaling	Y	N	Y	N
**Visualization**	Hierarchical plot	Y	N	N	N
	Circle plot	Y	N	Y	N
	Bubble plot	Y	N	N	Y
	Alluvial plot	Y	N	N	N
**Language**		R	R	R	Python
