# Supplementary material for: Inference and analysis of cell-cell communication using CellChat
Source: Nat Commun. 2021 Feb 17;12:1088. doi: 10.1038/s41467-021-21246-9 (PMC7889871; doi:10.1038/s41467-021-21246-9)
Supplement: Supplementary file 3 — Description of Additional Supplementary Files [file 41467_2021_21246_MOESM3_ESM.pdf]

**Description of Additional Supplementary Files**

File Name: Supplementary Data 1
Description: Supplementary excel file lists the inferred ligand-receptor pairs for each method (CellChat, CellPhoneDB, iTALK and SingleCellSignalR) between any two subpopulations from the four spatially colocalized cell populations in E14.5 embryonic mouse skin. The shared ligand-receptor pairs between CellChat and the other three methods are bolded in each result from the three methods.

File Name: Supplementary Data 2
Description: Supplementary excel file shows the average expressions of all predicted ligands and receptors in each cell subpopulation. Related to Figure 3 and Figure 5.
