# Supplementary material for: Inference and analysis of cell-cell communication using CellChat
Source: Nat Commun. 2021 Feb 17;12:1088. doi: 10.1038/s41467-021-21246-9 (PMC7889871; doi:10.1038/s41467-021-21246-9)
Supplement: Supplementary file 6 — Reporting Summary [file 41467_2021_21246_MOESM6_ESM.pdf]

nature research

Corresponding author(s): Qing Nie

Last updated by author(s): Dec 17, 2020

Reporting Summary

Nature Research wishes to improve the reproducibility of the work that we publish. This form provides structure for consistency and transparency in reporting. For further information on Nature Research policies, see our Editorial Policies and the Editorial Policy Checklist.

Statistics

For all statistical analyses, confirm that the following items are present in the figure legend, table legend, main text, or Methods section.

n/a	Confirmed	
☐	☒	The exact sample size (*n*) for each experimental group/condition, given as a discrete number and unit of measurement
☐	☒	A statement on whether measurements were taken from distinct samples or whether the same sample was measured repeatedly
☐	☒	The statistical test(s) used AND whether they are one- or two-sided<br>*Only common tests should be described solely by name; describe more complex techniques in the Methods section.*
☒	☐	A description of all covariates tested
☒	☐	A description of any assumptions or corrections, such as tests of normality and adjustment for multiple comparisons
☐	☒	A full description of the statistical parameters including central tendency (e.g. means) or other basic estimates (e.g. regression coefficient) AND variation (e.g. standard deviation) or associated estimates of uncertainty (e.g. confidence intervals)
☐	☒	For null hypothesis testing, the test statistic (e.g. *F*, *t*, *r*) with confidence intervals, effect sizes, degrees of freedom and *P* value noted<br>*Give P values as exact values whenever suitable.*
☒	☐	For Bayesian analysis, information on the choice of priors and Markov chain Monte Carlo settings
☒	☐	For hierarchical and complex designs, identification of the appropriate level for tests and full reporting of outcomes
☐	☒	Estimates of effect sizes (e.g. Cohen's *d*, Pearson's *r*), indicating how they were calculated

Our web collection on statistics for biologists contains articles on many of the points above.

Software and code

Policy information about availability of computer code

Data collection	All data analyzed within this manuscript are publicly available. No additional software was used for the data collection process.
Data analysis	We performed all the data analysis using our developed CellChat software (Version: 0.0.1) in this paper, which is available at GitHub (https://github.com/sqjin/CellChat). CellChat R package is built upon R 3.5. CellChat have many dependencies, including sna v2.5, igraph v1.2.5, ggplot2 v3.3.2, future v1.19.1, future.apply v1.6.0, pbapply v1.4-3, NMF v0.23.0, ggalluvial v0.12.2, dplyr v1.0.2, svglite v1.2.3, ggrepel v0.8.2, RColorBrewer v1.1-2, cowplot v1.1.0, ComplexHeatmap v2.7.1, RSpectra v0.16-0, Rcpp v1.0.5,RcppEigen v0.3.3, reticulate v1.16, FNN v1.1.3, shape v1.4.5.

For manuscripts utilizing custom algorithms or software that are central to the research but not yet described in published literature, software must be made available to editors and reviewers. We strongly encourage code deposition in a community repository (e.g. GitHub). See the Nature Research guidelines for submitting code & software for further information.

Data

Policy information about availability of data

All manuscripts must include a data availability statement. This statement should provide the following information, where applicable:

- Accession codes, unique identifiers, or web links for publicly available datasets
- A list of figures that have associated raw data
- A description of any restrictions on data availability

CellChatDB is included in the CellChat repository (https://github.com/sqjin/CellChat). KEGG pathway database is available at https://www.genome.jp/kegg/pathway.html. The datasets analyzed in this study are available from the Gene Expression Omnibus (GEO) repository under the following accession numbers: GSE113854 (https://www.ncbi.nlm.nih.gov/geo/query/acc.cgi?acc=GSE113854), GSE122043 (including four samples GSM3453535, GSM3453536, GSM3453537,

GSM3453538; https://www.ncbi.nlm.nih.gov/geo/query/acc.cgi?acc=GSE122043) and GSE147424 (https://www.ncbi.nlm.nih.gov/geo/query/acc.cgi?acc=GSE147424).

Field-specific reporting

Please select the one below that is the best fit for your research. If you are not sure, read the appropriate sections before making your selection.

☒ Life sciences ☐ Behavioural & social sciences ☐ Ecological, evolutionary & environmental sciences

For a reference copy of the document with all sections, see nature.com/documents/nr-reporting-summary-flat.pdf

Life sciences study design

All studies must disclose on these points even when the disclosure is negative.

Sample size	No sample size calculation was performed. All data used in this manuscript were taken from public resources and used to demonstrate the functionalities of CellChat. Choice of skin dataset was determined by our prior expertise on the aspects of skin morphogenesis and regeneration, its complex cellular make-up and the fact that the role of many signaling pathways in skin is well-established, which enables meaningful literature-based interpretation of a portion of CellChat predictions. We apply CellChat to three mouse skin datasets (Day 12 wound healing dataset, E13.5 embryonic development dataset and E14.5 embryonic development dataset), and one human skin dataset. The two mouse embryonic skin datasets are used to studying temporal changes of intercellular communications in the same tissue, and the wound healing dataset is used to show the communication in vastly distinct biological contexts – embryonic morphogenesis vs. wound-induced repair. The human dataset is used to show CellChat's ability in analyzing human dataset. Therefore, it is sufficient to demonstrate the functionalities of CellChat.
Data exclusions	We performed quality control of single-cell RNA-seq data based on the common used and pre-established criteria in this field. Low quality cells were removed based on the amount of UMI count, the number of genes and the fraction of mitochondrial counts. Genes were removed if they were expressed in less than 200 cells.
Replication	All Attempts at replication were successful and can be performed independently
Randomization	The allocation was random
Blinding	All results are based on published data which have been studied in their original publications. Therefore, blinding from investigators is not possible when we reanalyzed the data. Group allocation information was never provided to the computational algorithms.

Reporting for specific materials, systems and methods

We require information from authors about some types of materials, experimental systems and methods used in many studies. Here, indicate whether each material, system or method listed is relevant to your study. If you are not sure if a list item applies to your research, read the appropriate section before selecting a response.

Materials & experimental systems

n/a	Involved in the study	
☒	☐	Antibodies
☒	☐	Eukaryotic cell lines
☒	☐	Palaeontology and archaeology
☒	☐	Animals and other organisms
☒	☐	Human research participants
☒	☐	Clinical data
☒	☐	Dual use research of concern

Methods

n/a	Involved in the study	
☒	☐	ChIP-seq
☒	☐	Flow cytometry
☒	☐	MRI-based neuroimaging
